# Heterologous Aggregates Promote *De Novo* Prion Appearance via More than One Mechanism

**DOI:** 10.1371/journal.pgen.1004814

**Published:** 2015-01-08

**Authors:** Fatih Arslan, Joo Y. Hong, Vydehi Kanneganti, Sei-Kyoung Park, Susan W. Liebman

**Affiliations:** 1 Department of Biological Sciences, University of Illinois at Chicago, Chicago, Illinois, United States of America; 2 Department of Biochemistry and Molecular Biology, University of Nevada, Reno, Nevada, United States of America; Washington University School of Medicine, United States of America

## Abstract

Prions are self-perpetuating conformational variants of particular proteins. In yeast, prions cause heritable phenotypic traits. Most known yeast prions contain a glutamine (Q)/asparagine (N)-rich region in their prion domains. [*PSI^+^*], the prion form of Sup35, appears *de novo* at dramatically enhanced rates following transient overproduction of Sup35 in the presence of [*PIN^+^*], the prion form of Rnq1. Here, we establish the temporal *de novo* appearance of Sup35 aggregates during such overexpression in relation to other cellular proteins. Fluorescently-labeled Sup35 initially forms one or a few dots when overexpressed in [*PIN^+^*] cells. One of the dots is perivacuolar, colocalizes with the aggregated Rnq1 dot and grows into peripheral rings/lines, some of which also colocalize with Rnq1. Sup35 dots that are not near the vacuole do not always colocalize with Rnq1 and disappear by the time rings start to grow. Bimolecular fluorescence complementation failed to detect any interaction between Sup35-VN and Rnq1-VC in [*PSI* ^+^][*PIN* ^+^] cells. In contrast, all Sup35 aggregates, whether newly induced or in established [*PSI* ^+^], completely colocalize with the molecular chaperones Hsp104, Sis1, Ssa1 and eukaryotic release factor Sup45. In the absence of [*PIN^+^*], overexpressed aggregating proteins such as the Q/N-rich Pin4C or the non-Q/N-rich Mod5 can also promote the *de novo* appearance of [*PSI* ^+^]. Similar to Rnq1, overexpressed Pin4C transiently colocalizes with newly appearing Sup35 aggregates. However, no interaction was detected between Mod5 and Sup35 during [*PSI^+^*] induction in the absence of [*PIN* ^+^]. While the colocalization of Sup35 and aggregates of Rnq1 or Pin4C are consistent with the model that the heterologous aggregates cross-seed the *de novo* appearance of [*PSI* ^+^], the lack of interaction between Mod5 and Sup35 leaves open the possibility of other mechanisms. We also show that Hsp104 is required in the *de novo* appearance of [*PSI^+^*] aggregates in a [*PIN* ^+^]-independent pathway.

## Author Summary

Certain proteins can misfold into β-sheet-rich, self-seeding aggregates. Such proteins appear to be associated with neurodegenerative diseases such as prion, Alzheimer's and Parkinson's. Yeast prions also misfold into self-seeding aggregates and provide a good model to study how these rogue polymers first appear. *De novo* prion appearance can be made very frequent in yeast by transient overexpression of the prion protein in the presence of heterologous prions or prion-like aggregates. Here, we show that the aggregates of one such newly induced prion are initially formed in a dot-like structure near the vacuole. These dots then grow into rings at the periphery of the cell prior to becoming smaller rings surrounding the vacuole and maturing into the characteristic heritable prion tiny dots found throughout the cytoplasm. We found considerable colocalization of two heterologous prion/prion-like aggregates with the newly appearing prion protein aggregates, which is consistent with the prevalent model that existing prion aggregates can cross-seed the *de novo* aggregation of a heterologous prion protein. However, we failed to find any physical interaction between another heterologous aggregating protein and the newly appearing prion aggregates it stimulated to appear, which is inconsistent with cross-seeding.

## Introduction

Prions were first described as self-perpetuating infectious agents devoid of nucleic acids that cause several fatal neurodegenerative diseases. Prion diseases, also known as transmissible spongiform encephalopathies (TSEs), were shown to infect a variety of mammals [1]. All known mammalian prion diseases are caused by conversion of largely α-helical cellular prion protein PrP^C^ into fibrous β-sheet-rich ordered aggregates (amyloids) called PrP^Sc^ (associated with scrapie) [2]. Curiously, PrP^Sc^ can exist in different heritable forms, called strains, which cause neurodegenerative diseases with different characteristics and pathologies [3]–[5].

A number of other neurodegenerative diseases are also associated with conversion of a soluble protein to amyloid. For example, amyloid-like forms of Aβ and Tau, α-synuclein, huntingtin, FUS/TLS, TDP-43 or SOD1 are linked respectively to Alzheimer's (AD) [6], Parkinson's (PD) [7], [8], Huntington's (Htt) [9] and Amyotrophic Lateral Sclerosis (ALS) diseases [10]–[15]. Factors that influence the spontaneous conversion to amyloid are of considerable interest as possible disease risk factors. One important finding is that heterologous amyloid can promote the *de novo* conversion of a protein to amyloid. For example, Aβ accelerated the *in vivo* aggregation of tau [16], and Aβ and α-synuclein seeded each other's aggregation *in vitro* [17]. Indeed, recently distinct conformational variants of α-synuclein aggregates were shown to differentially promote the aggregation of tau in neurons [18].

Several proteins in the simple eukaryote yeast have been shown to convert from soluble to amyloid. The amyloid forms of these proteins are self-propagating prions associated with transmissible phenotypes [19]–[28]. These proteins provide good models for the amyloid conversion of human disease proteins.

For both human and yeast proteins, only a portion of the protein, called the prion domain (PrD), converts to amyloid. This portion of the protein is required for prion induction and propagation [29], [30]. The PrD of most known yeast prions is glutamine (Q) and asparagine (N) rich. Likewise, several human aggregating disease proteins e.g. huntingtin, TDP-43 and FUS contain Q/N-rich regions [31], [32]. In contrast, the recently discovered yeast [*MOD^+^*] prion, composed of Mod5, as well as PrP, Aβ and α-synuclein do not contain Q/N rich domains [27]. Similar to the mammalian PrP strains, yeast prions can also fold into numerous heritable conformations, called variants, leading to different degrees of altered phenotypes [33]–[36].

The most well-studied yeast prion is [*PSI^+^*], the prion form of Sup35. In its native form, Sup35 (release factor 3) works with Sup45 (release factor 1) to promote translational termination at stop codons [37]. The Sup35 protein consists of three major domains: N-proximal (N domain) required for prion induction and propagation; a highly charged middle (M) domain conferring solubility to the molecule and containing Hsp104 binding sites [38] and a C-terminal (C) domain essential for translational termination and viability [33], [39]–[41]. [*PSI^+^*] forms when Sup35 molecules assemble into amyloid-like aggregates, causing loss-of-function in translation termination, which leads to read-through of stop codons [20], [42], [43].

The spontaneous appearance of prions in yeast is extremely rare. Indeed, the conversion of prion-free cells, [*psi^-^*] to [*PSI^+^*] was determined to be ∼5.8×10^−7^ [44]–[47]. On the other hand, overproduction of full-length Sup35 or its prion containing domain (Sup35NM) can increase the *de novo* appearance of [*PSI^+^*] dramatically, presumably by increasing the chance of Sup35 prion domains to misfold and interact [48]. This enhanced formation of [*PSI^+^*] requires either the presence of another prion [22], [49], [50] or the simultaneous overexpression of heterologous Q/N-rich domains [22], [51]. The best studied example of this stimulation by a prion involves [*PIN^+^*], the prion form of the Rnq1 protein. Although [*PIN^+^*] dramatically enhances the appearance of [*PSI^+^*], it is not required for [*PSI^+^*] propagation [49].

Understanding how [*PIN^+^*] enhances *de novo* induction of [*PSI^+^*] will help us understand analogous interactions between heterologous human disease proteins. Several models have been proposed (reviewed in [52]). The cross-seeding model suggests that [*PIN^+^*] initially acts as a seed for the conversion of the Sup35 prion domain into the [*PSI^+^*] conformation. Once [*PSI^+^*] is established, it is proposed to create its own seeds independent of [*PIN^+^*], allowing it to continue to propagate efficiently [53]. Several *in vitro* studies provide evidence in favor of induction of [*PSI^+^*] via cross-seeding [52], [54], and for the enhanced rate of polymerization of other proteins in the presence of heterologous aggregates [55]–[61]. Most notably, mCherry:FUS fibers were extended in length heterotypically when exposed to monomeric GFP:hnRNPA1 [55]. Definitive evidence for cross-seeding *in vivo* is much more difficult to obtain. Still, a fusion of the prion domain of Sup35 (NM) and Rnq1 lead to the efficient induction of [*PSI^+^*] in the presence of [*PIN^+^*], even without Sup35 overexpression [62], presumably because the fusion efficiently brings Sup35NM to the Rnq1 aggregates, thereby increasing the chance of physical association and resulting cross-seeding. Also, different [*PIN^+^*] variants preferentially cause the genesis of different variants of [*PSI* ^+^] [63], [64], which can be easily explained by cross-seeding but not by chaperone titration.

The titration model postulates that cellular factors responsible for the disassembly of aggregates and the refolding of misfolded proteins are so busy working on the existing [*PIN^+^*] prion that they are not available to prevent the appearance of the new prion, [*PSI^+^*] [22], [51], [65]. In support of this model, prion-like aggregates have been shown to colocalize with chaperones, reducing the cytosolic level of chaperones and thereby affecting the stability of heterologous prion aggregates in the cell [66]–[70].

Molecular chaperones, which are normally involved in proper protein folding play a critical role in the maintenance of yeast prions (reviewed in [71]). Particularly, the Hsp104 chaperone in conjunction with chaperones of the Hsp70 (Ssa/Ssb) and Hsp40 (Sis1) families was shown to shear prion aggregates into smaller fragments that promote fiber growth and transmission to daughter cells [72]–[77]. The shearing activity of Hsp104 is antagonized by millimolar concentrations of guanidine hydrochloride (GuHCl), leading to the loss of [*PSI^+^*] [78], [79], and other yeast prions [19], [50]. Hsp104 is required for the propagation of almost all known yeast prions [72], [80]–[82].

Stimulation of *de novo* generation of prions in yeast is achieved by inducing overexpression of the corresponding prion protein. The resulting aggregates have been monitored with fluorescent derivatives. The *de novo* induction of [*PSI^+^*] promoted by [*PIN^+^*] was shown to display various Sup35 aggregates and go through several stages. Overexpression of Sup35NM-GFP, gave rise to fluorescent dot, line and ring-like assemblies [83]–[85]. The fluorescent rings induced by Sup35NM-GFP overexpression is a hallmark of [*PSI^+^*] induction. Indeed, most viable ring/dot-bearing cells gave rise to [*PSI^+^*] progeny [83]–[86]. Sup35 dots appeared earlier than rings and lines [84]. Ring-like aggregates were shown to be first peripheral along the cell membrane, and later internal surrounding the vacuole [83], [85]. When cells with such rings were followed in media that turned down Sup35 overexpression, Sup35NM-GFP appeared as dots in daughter cells, a typical feature of [*PSI^+^*] [83]–[85]. Once [*PSI^+^*] is established, Sup35NM-GFP overexpression results in one or a few large mature dots, or clumps but rings do not appear at all [29], [43]. These large dots replace the numerous small Sup35-GFP aggregates seen in [*PSI^+^*] cells with endogenous Sup35 tagged with GFP prior to overexpression [75]. When Sup35NM-GFP was constitutively overproduced in [*PIN^+^*] cells with a deletion of the Sup35 prion domain, only internal rings were observed prior to the transition to mature dots [87].

In this study, we report that the *de novo* appearance of [*PSI^+^*] aggregates begins with dots that co-localize with the main Rnq1 aggregate near the vacuole, that grow into peripheral rings and lines prior to the appearance of internal rings. Our studies also reveal preferential colocalization of Rnq1 and Pin4C aggregates with newly appearing vs. established [*PSI^+^*] aggregates, which is consistent with the cross-seeding model for [*PSI^+^*] induction. However, the failure of Mod5 to physically interact with Sup35 during Mod5-promoted [*PSI^+^*] induction suggests that cross-seeding is not involved. Finally, we provide evidence for the [*PIN* ^+^]-independent requirement of Hsp104 during [*PSI^+^*] induction *in vivo*.

## Results

### Newly induced Sup35 aggregates arise near the vacuole and then grow into peripheral rings

**Figure 1:**
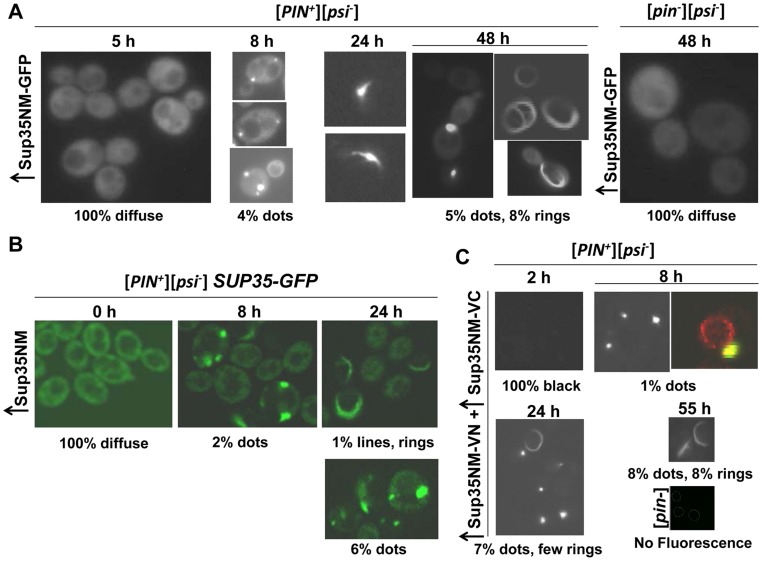
Sup35 forms vacuole-associated dots first, then rings during [*PSI^+^*] induction. **A.** Visualization of Sup35 aggregates induced by Sup35NM-GFP in [*PIN^+^*] cells. 74D-694 [*PIN^+^*][*psi^-^*] and [*pin^-^*][*psi^-^*] cells were grown in inducing media (2% Gal) to overexpress Sup35NM-GFP (p1951, see Table 5 for plasmid nomenclature). From the time of induction, Sup35NM-GFP signals were visualized with fluorescent microscope over a time course (detailed in Table 1). Percentages are based on n≈500-800. **B.** Visualization of Sup35 aggregates induced by untagged Sup35NM in [*PIN^+^*] cells with endogenous *SUP35-GFP*. [*PIN^+^*] cells with endogenous *SUP35* labeled with GFP (L3107) were grown in 2% Gal to overexpress untagged Sup35NM (p2036). High levels of Sup35NM induced endogenous Sup35-GFP to form dots and rings at the indicated times (n≈600). **C.** Visualization of Sup35 aggregates induced by Sup35NM BiFC constructs in [*PIN^+^*] cells. Sup35NM-VC (p1892) and Sup35NM-VN (p1893) in 74D-694 [*PIN^+^*][*psi^-^*] cells were co-overexpressed by growth in 0.2% Gal. The picture on the right (magnified ∼3X) at 8 h represents FM4-64 stained (red) cells with Sup35 BiFC dot (yellow). BiFC fluorescence was detected with a YFP filter. (n≈600).

We used GFP-tagged SUP35 constructs to visualize the initial conversion of Sup35 from soluble to aggregated protein when [*psi^-^*] cells were induced to become [*PSI^+^*] by overexpressing Sup35(NM). Sup35NM-GFP overexpressed in [*PIN^+^*][*psi^-^*] cells (Fig. 1A) progressed over time from diffuse cytoplasmic fluorescence in all cells to some cells with one to three fluorescent foci one of which was always near the vacuole, to more cells with dots. Later, peripheral rings and lines started to replace dots in some cells (see Table 1 for details). In contrast, [*pin^-^*] cells showed no aggregates of Sup35NM-GFP at any time point. Even when we dramatically reduced the level of Sup35NM-GFP overexpression by growing cells in 0.2 rather than 2% Gal, the type and order of appearance of these aggregates did not change (S1 Table).

**Table 1:** Quantitative data for the aggregation of fluorescently-tagged Sup35 shown in Fig. 1.

Experiment	Observations following Sup35 overexpression for 1–3 days
Fig. 1A	**5 h**: All diffuse
	**8 h**: 4% 1–3 foci one of which is near the vacuole
	**16 h**: 7% dots
	**24 h**: peripheral rings and lines started to replace dots in some cells
	**48 h**: 8% peripheral rings/lines and 5% dots
Fig. 1B and S1A	**0 h**: All diffuse
	**8 h**: 2% 1–3 foci one of which appears near the vacuole
	**16 h**: 4% dots
	**24 h**: 1% rings/lines and 6% dots
Fig. 1C	**2 h**: All black
	**8 h**: 1% dots near the vacuole
	**24 h**: peripheral rings/lines and more dots
	**55 h**: 8% rings/lines; 8% dots

74D-694 [*PIN^+^*][*psi^-^*] cells were grown in 2% Gal to overexpress Sup35NM-GFP (from p1951) (Fig. 1A); or in 0.2% Gal to co-overexpress Sup35NM-VN (from p1893) and Sup35NM-VC (from p1892) (Fig. 1C). 74D-694 [*PIN^+^*][*psi^-^*] *SUP35-GFP* cells were grown in 2% Gal to overexpress Sup35NM (from p2036) (in Figure 1B) or Sup35 (from p743) (in S1A Fig.).

In another approach, we examined endogenous Sup35 tagged with GFP when untagged Sup35NM was overexpressed in [*PIN^+^*][*psi^-^*] cells (Fig. 1B, Table 1). Upon induction of Sup35NM, all cells initially showed diffuse Sup35-GFP fluorescence, which was later seen as cytoplasmic dots one of which was near the vacuole and then, lines and rings in some cells. Similar results were observed when untagged full-length Sup35 was overexpressed (S1A Fig.). As expected, [*pin^-^*] cells always displayed diffuse Sup35-GFP molecules in the presence of Sup35NM overexpression (S1B Fig.).

Because the diffuse fluorescence of Sup35NM-GFP observed in the experiments above might have masked the visualization of initial Sup35 aggregates during [*PSI^+^*] induction, we overexpressed Sup35NM from Bimolecular Fluorescence Complementation [88], [89] (BiFC) constructs, Sup35NM-VN and Sup35NM-VC, simultaneously in [*PIN^+^*][*psi^-^*] cells (Fig. 1C, Table 1). Prior to 8 h of induction, no fluorescence was detected. The lack of diffuse fluorescence suggests that Sup35 aggregation does not begin all over the cell. At 8 h, a few cells showed fluorescent dots near the vacuole revealed by FM4-64 staining [90], but no lines/rings were visible. By 24-55 h, peripheral rings and lines appeared and more cells displayed dots. In control [*pin^-^*] cells, no fluorescence was detected.

**Figure 2:**
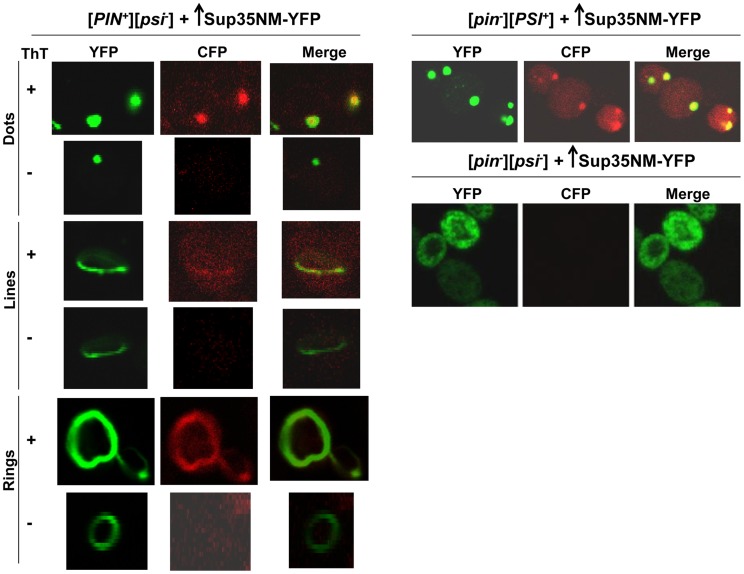
Sup35 newly induced aggregates are not always amyloid-like, but mature Sup35 aggregates are. [*PIN^+^*][*psi^-^*], [*pin^-^*][*PSI^+^*], [*pin^-^*][*psi^-^*] yeast cells overexpressing YFP-fusions of Sup35NM (p1753) for 24 h in 2% Gal were stained with Thioflavin T to assess amyloid formation. Sup35NM-YFP inclusions in [*pin^-^*][*PSI^+^*] contained amyloid (ThT fluorescence signal in CFP channel), however in [*pin^-^*][*psi^-^*], there were no inclusions formed, and these cells were negative in ThT fluorescence. In [*PIN^+^*][*psi^-^*], overexpression of Sup35NM-YFP induced the formation of dots, lines and rings, which were sometimes but not always ThT-positive. From top to bottom, pictures represent ThT positive and negative, respectively, for dots, lines and rings.

To determine if the dots and rings/lines that appeared within 24 h of induction of Sup35NM-YFP overexpression show amyloid-like properties, we stained the newly appearing Sup35NM-YFP aggregates in [*PIN^+^*][*psi^-^*] cells with Thioflavin T (ThT) (Fig. 2): 30% of the dots and 60% of the rings were ThT-positive. As expected, Sup35NM-YFP mature dots in [*pin^-^*][*PSI^+^*] were all stained with ThT. In control [*pin^-^*][*psi^-^*] cells however, diffuse Sup35NM-YFP fluorescence did not show any ThT fluorescence. These data suggested that Sup35NM-YFP does not always form amyloidogenic aggregates during [*PSI^+^*] induction, but eventually becomes amyloid in mature [*PSI^+^*].

**Figure 3:**
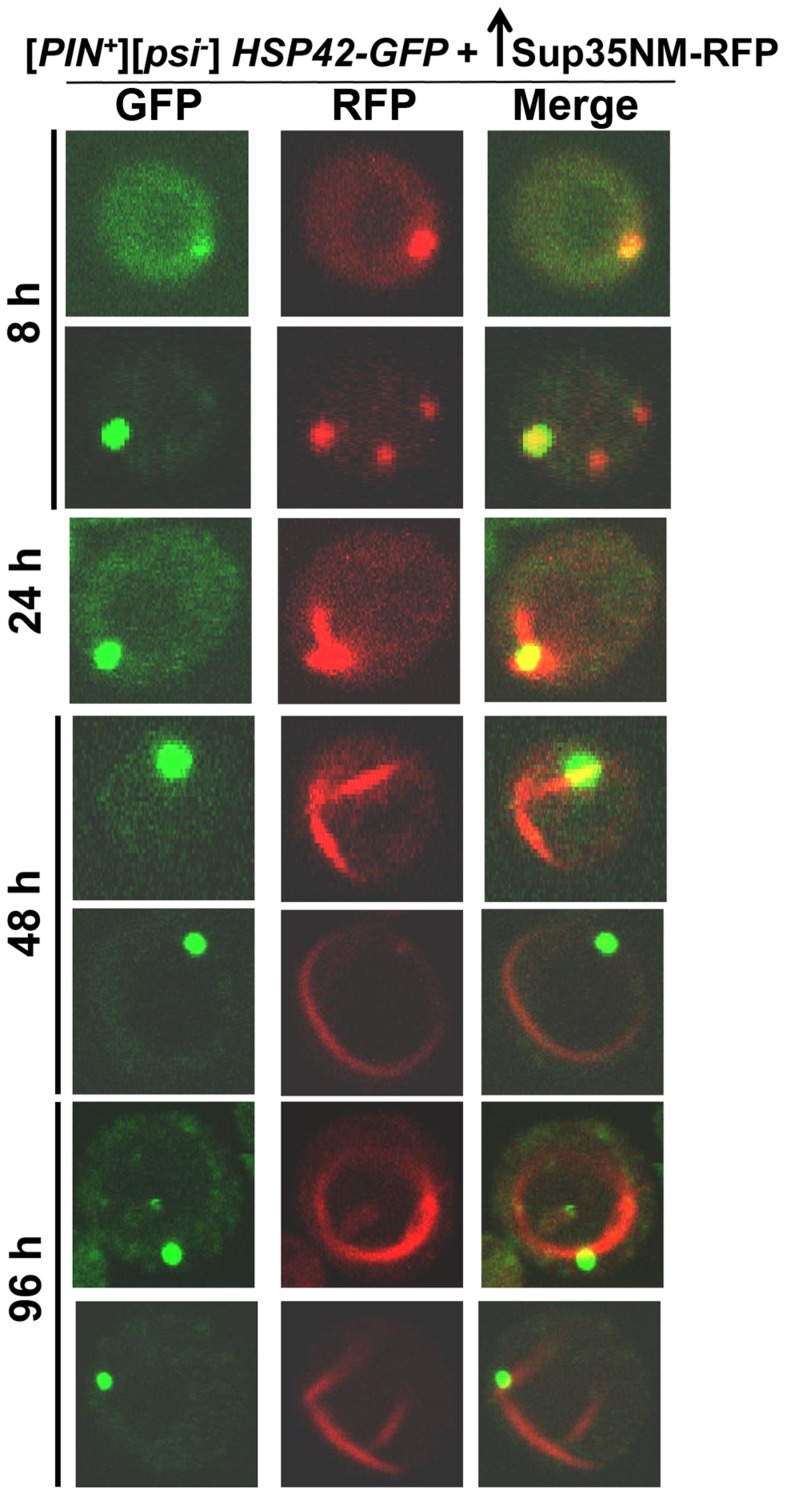
Sup35 aggregates initially appear near the vacuole, from which short lines extend to the periphery to form rings. Sup35NM-RFP was overexpressed from p2017 by growing [*PIN^+^*] BY4741 cells with endogenous *HSP42-GFP* in 2% Gal. Time shown is after the addition of 2% Gal (see details in Table 2).

To further investigate the subcellular localization of the initial Sup35 dots, we used BY4741 cells with genomic *HSP42* tagged with GFP [91] (see Table 2 for details). Hsp42 is a small heat shock protein that appears as one big dot near the vacuole, sometimes referred to as the IPOD for the site(s) of deposit of insoluble protein aggregates [92]–[94]. Overexpression of Sup35NM-RFP in [*PIN^+^*][*psi^-^*] *HSP42-GFP* cells first caused the occasional appearance of cells with 1-6 dots, one of which always colocalized with the Hsp42-GFP dot (Fig. 3). Later, in some cells, Sup35NM-RFP fluorescence extended from a bright dot that colocalized with the Hsp42-GFP dot as short lines tangent to the vacuole or as lines extending to the cell periphery. Interestingly, the multiple Sup35NM-RFP dots observed initially were never seen later once lines appeared, suggesting that Sup35NM-RFP aggregates that did not colocalize with Hsp42-GFP were solubilized, or may have joined the lines. Eventually, in some cells, Sup35 formed internal rings surrounding the vacuole as seen previously [83], [84], intersecting the Hsp42-GFP dot, and in a very few cells, lines were seen to extend from the Hsp42-GFP dot peripherally and around the vacuole simultaneously.

**Table 2:** Data for the aggregation of Sup35NM-RFP in *HSP42-GFP* cells shown in Fig. 3.

Experiment	Observations following Sup35 overexpression for 4 days
Fig. 3	**8 h**: 4% single dot and 2.5% 3–6 dots
	**24 h**: 3% short lines
	**48 h**: 4.5% rings/lines
	**96 h**: 3% internal rings and <0.1% lines around the vacuole and extending to the periphery

BY4741 [*PIN^+^*][*psi^-^*] *HSP42-GFP* cells were grown in 2% Gal to overexpress Sup35NM-RFP (from p2017). Data for the florescence of only Sup35NM-RFP is tabulated. Hsp42-GFP was always seen as 1 big spot near the vacuole in all cells.

To determine the localization of Sup35 newly induced aggregates with respect to the vacuole, we overexpressed Sup35NM-RFP in [*PIN^+^*] cells with genomic *VPH1* tagged with GFP (S2 Fig.). Vph1 is a subunit of the vacuolar-ATPase protein and marks the vacuolar membrane [95]. We found that Sup35 early dots (after 24 h of Sup35NM-RFP overexpression) were localized near the vacuole, and later, short lines extended outward from the vacuole to the periphery of the cell. Then, as expected, Sup35 formed peripheral rings, and eventually perivacuolar rings.

In summary, the various experiments above showed that during the *de novo* aggregation of Sup35 induced by its overexpression, Sup35 initially formed dots, one of which perfectly colocalized with the Hsp42-GFP dot near the vacuole. Then, Sup35 lines extended from this dot to form peripheral and eventually perivacuolar rings, while the other initial Sup35 dots disappeared.

### Colocalization of Rnq1 with Sup35 dots

**Figure 4:**
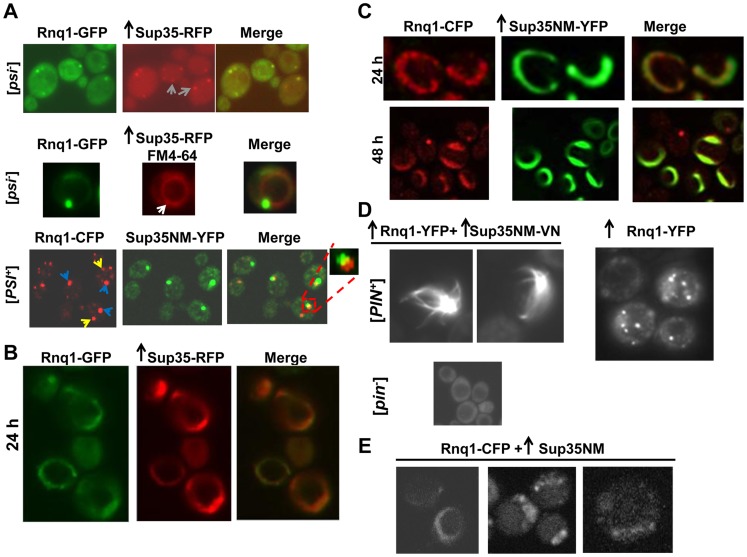
Rnq1 aggregates colocalize with Sup35 aggregates when [*PSI^+^*] is induced *de novo*. **A.** Colocalization of Sup35 newly appearing vs. mature [*PSI^+^*] dots with normal levels of Rnq1. Sup35-RFP was overexpressed (p1678) in [*PIN^+^*][*psi^-^*] cells with the plasmid p1730 expressing Rnq1-GFP from its own promoter by growth in 2% Gal (top). Gray arrows show additional Sup35 dots that did not overlap Rnq1 and that are away from the vacuole. The middle panel (magnified ∼2X) shows an Rnq1-GFP dot colocalized with Sup35-RFP dot (white arrow) located near the vacuole stained with FM4-64 (red circle). [*PIN^+^*] cells with established [*PSI^+^*] were grown in 0.05% Gal for 3–4 h to stain mature [*PSI^+^*] dots marked by Sup35NM-YFP (bottom). These dots showed partial colocalization (enlarged box) with [*PIN^+^*] dots marked by Rnq1-CFP. Blue arrows indicate partially colocalized Rnq1 dots; yellow arrows indicate non-colocalized additional Rnq1 dots. **B.** Colocalization of Sup35 rings with normal levels of Rnq1 expressed from a plasmid. Sup35-RFP was overexpressed from p1678 in [*PIN^+^*][*psi^-^*] cells with the *CEN* plasmid p1730 expressing Rnq1-GFP from its own promoter by growth in 2% Gal. Sup35-RFP rings colocalized with Rnq1-GFP rings after 24 h. **C.** Colocalization of Sup35 rings with normal levels of Rnq1 expressed from an integrated construct. Rnq1 tagged with CFP, integrated into genomic *TRP1* was expressed from its own promoter in [*PIN^+^*] cells overexpressing Sup35NM-YFP from p1753, by growth in 2% Gal for the indicated times. **D.** Visualization of [*PIN^+^*] aggregates during [*PSI^+^*] induction with overexpressed Rnq1 and Sup35. Rnq1-YFP was co-overexpressed with Sup35NM respectively from p1728 and p1893, by growth in 2% Gal for 24 h. Rnq1-YFP formed lines and mesh-like aggregates in the presence of [*PIN^+^*] and Sup35NM overexpression, but remained diffuse in [*pin^-^*]. In the absence of Sup35NM overexpression, [*PIN^+^*] contained only multiple dots of Rnq1-YFP. **E.** Visualization of [*PIN^+^*] aggregates during Sup35 overexpression with normal levels of Rnq1. Untagged Sup35NM (p2036) was overexpressed in [*PIN^+^*] *RNQ1-CFP* integrants in 2% Gal for 48 h. Rnq1-CFP displayed ring/line-like structures in the presence of Sup35NM overexpression in 10% of [*PIN^+^*] cells (4 trials, each with n≈350).

To visualize the relationship of Sup35 and Rnq1 during the *de novo* induction of [*PSI^+^*], we expressed Rnq1-GFP under its own promoter, and overproduced Sup35-RFP in [*PIN^+^*][*psi^-^*] cells (Fig. 4A, top). Sup35-RFP initially formed fluorescent dots but no lines or rings (Details in S2 Table). All the Rnq1-GFP dots perfectly colocalized with Sup35-RFP dots, but only 60% of the Sup35-RFP dots colocalized with Rnq1-GFP foci. Interestingly, the colocalized Rnq1-Sup35 dots were always associated with the vacuole revealed by FM4-64 staining (Fig. 4A, middle). Additional Sup35 dots that did not overlap Rnq1 were away from the vacuole (Fig. 4A, gray arrows).

These Sup35 dots have different characteristics from Sup35 dots seen in mature [*PSI^+^*] cells. For example, while the results above showed that newly appearing vacuole-associated Sup35 aggregates perfectly colocalize with Rnq1, mature Sup35 aggregates in established [*PSI^+^*] cells did not entirely overlap Rnq1 (seen as in two sets intersecting in a Venn diagram) (Fig. 4A, bottom). Also, additional Rnq1-CFP dots existed in [*PIN^+^*][*PSI^+^*] cells that did not show any colocalization with Sup35 (Fig. 4A bottom, arrows, enlarged box).

In summary, after 6 h of [*PIN^+^*]-promoted Sup35-RFP aggregation, all Rnq1 dots perfectly overlapped newly induced Sup35 dots around the vacuole, but additional Sup35 dots (away from the vacuole) without overlapping Rnq1 sometimes existed in those cells. In contrast, in established [*PSI^+^*] cells, all Sup35 dots partially overlapped Rnq1 dots, but additional Rnq1 dots without overlapping Sup35 also existed.

### Colocalization of Rnq1 with Sup35 rings

To look for colocalization of Rnq1 with Sup35 rings, we induced overexpression of Sup35-RFP for 24 h in [*PIN^+^*] cells also expressing Rnq1-GFP from its own promoter. Sup35-RFP formed rings 70% of which colocalized with Rnq1-GFP (Fig. 4B). In the remaining 30% of cells with Sup35-RFP rings that did not colocalize, Rnq1-GFP fluorescence was instead diffuse or in the form of dots (S3A Fig., S3 Table). In contrast, essentially all Rnq1-GFP rings colocalized with Sup35-RFP rings (S4 Table). As expected, Rnq1-GFP and Sup35-RFP always remained diffuse in [*pin^-^*] cells (S3B Fig.).

In another version of this experiment, Sup35NM-YFP was overexpressed in [*PIN^+^*][*psi^-^*] cells expressing Rnq1-CFP under its own promoter. Sup35NM-YFP overexpressed for 24 h formed fluorescent dots or rings respectively, in 7 and 0.8% of the cells. In these cells, 90% of Sup35NM-YFP dots colocalized with Rnq1-CFP dots, and all Sup35NM-YFP rings colocalized with Rnq1-CFP rings (Fig. 4C top, S5 Table). Curiously, these Rnq1-CFP rings looked like beads on a string, rather than an uninterrupted full ring.

After 48 h of Sup35NM-YFP overexpression, Sup35NM-YFP formed rings in 11% of the cells, and 75% of these Sup35NM-YFP rings colocalized with Rnq1-CFP rings (Fig. 4C bottom). In the remaining 25% of cells with Sup35NM-YFP rings, Rnq1-CFP showed diffuse or dot fluorescence (S6 Table). On the other hand, essentially all Rnq1-CFP rings colocalized with Sup35NM-YFP rings (S7 Table). In control experiments, [*pin^-^*] cells never formed any aggregates when Sup35NM-YFP was overexpressed (S4A Fig.). [*PIN^+^*] cells showed only Rnq1-CFP dots, but no lines/rings when Sup35NM-YFP expression remained repressed in 2% Glucose (S4B Fig.) and when cells with empty vector expressing YFP were grown in 2% Gal (S4C Fig.). These colocalization data were based on visually checking different planes of the cells by moving the focal plane up and down, and were also confirmed by collecting z-stacks from representative cells (S5 Fig.).

Next, we co-overexpressed Rnq1-YFP and Sup35NM-VN in [*PIN^+^*][*psi^-^*] cells (Fig. 4D). In 24 h, 7% of the cells had one Rnq1-YFP dot with lines extending from it in all directions, referred to as mesh-like aggregates. In controls, when Rnq1-YFP was overexpressed in [*PIN^+^*] without overexpressing Sup35NM, 90% of the cells showed multiple fluorescent dots, 10% had diffuse fluorescence, and none had mesh-like aggregates. Also, overexpressing Rnq1-YFP and Sup35NM-VN simultaneously in [*pin^-^*] control cells did not result in any aggregate formation. When cells from cultures with Rnq1-YFP mesh-like aggregates (7%) were scored for [*PSI^+^*], 6.5% of these cells formed pink or white colonies and were able to grow on media lacking adenine (SD-Ade), indicative of [*PSI^+^*] (See Methods).

To visualize if Sup35 and Rnq1 have a close physical interaction during [*PSI^+^*] induction, we co-overexpressed Sup35NM-VN and Rnq1-VC in [*PIN^+^*][*psi^-^*] cells (S6 Fig., S8 Table). Initially (16 h post induction) 1.8% of the cells showed dots, but no lines/rings; but later (40 h post induction) peripheral rings, lines and mesh-like aggregates appeared and more cells displayed dots. We also co-overexpressed Sup35NM-VN and Rnq1-VC in established [*PSI^+^*] as well as in [*pin^-^*][*psi^-^*] cells and did not observe any fluorescence. These data indicate that Rnq1 and Sup35 interact in a close proximity during [*PSI^+^*] induction, but not in established [*PSI^+^*].

As expected, overexpression of untagged Sup35NM caused Rnq1-CFP expressed from its own promoter to align in ring/line-like aggregates in 10% of the [*PIN^+^*] cells (Fig. 4E). The Rnq1-CFP lines looked like beads on a string as seen previously in the presence of overexpressed YFP-tagged Sup35NM (Fig. 4C top). Such beads on a string never appeared in [*PIN^+^*] cells without overexpressed Sup35NM, where Rnq1-CFP fluorescent dots remained dispersed, or in [*pin^-^*], where Rnq1-CFP remained diffuse (S7 Fig.). When cells from the culture with Rnq1-CFP beads on a string (10%) were scored for [*PSI^+^*], 11% of these cells formed pink or white colonies, indicative of [*PSI^+^*].

The above experiments show that during the *de novo* aggregation of Sup35 in [*PIN^+^*] cells, overexpression of Sup35NM induced Rnq1 to form mesh-like or line/ring-like aggregates. Essentially all Rnq1 line/rings perfectly overlapped Sup35 line/rings, while some Sup35 line/rings did not overlap Rnq1. BiFC between Rnq1 and Sup35 confirmed that they form a close physical interaction during the induction of [*PSI^+^*], and initially form dots, and then lines/meshes; while they do not form such an interaction in established [*PSI^+^*].

### Localization of other proteins during [*PSI^+^*] induction

**Figure 5:**
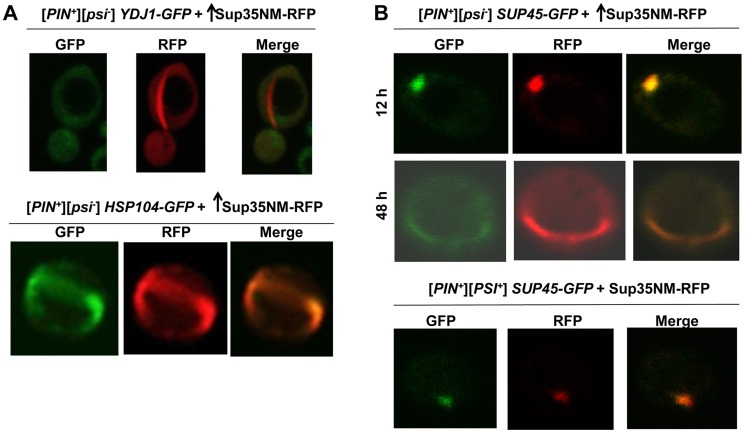
Induced Sup35 rings do not colocalize with Ydj1, but do colocalize with Hsp104 and Sup45. **A.** Colocalization of newly induced Sup35 aggregates with chaperones. Sup35NM-RFP was overexpressed (p2018) in BY4741 [*PIN^+^*] cells with GFP-tagged chaperones in 2% Gal for 48 h. While 9.3% of the cells contained Sup35NM-RFP peripheral rings and dots, Ydj1-GFP always remained cytoplasmically diffuse (n≈450) (top). In *HSP104-GFP* cells, by 48 h, Hsp104-GFP formed rings in 5.6% of the [*PIN^+^*] cells all of which colocalized with Sup35 rings during [*PSI^+^*] induction. (n≈350) (bottom). **B.** Colocalization of newly induced vs. established Sup35 aggregates with Sup45. Sup35NM-RFP was overexpressed from p2018 in [*PIN^+^*] cells with endogenous *SUP45* tagged with GFP by growth in 2% Gal for the indicated times. Although overexpression of Sup45 inhibits [*PSI^+^*] induction [107], Sup45-GFP perfectly colocalized with Sup35NM-RFP aggregates in [*PIN^+^*][*psi^-^*] cells (top, n≈400). After these cells were allowed to propagate [*PSI^+^*], Sup45-GFP also perfectly overlapped Sup35 mature dot (bottom).

In order to determine what other proteins (particularly chaperones) colocalize with Sup35 during its aggregation in the presence of [*PIN^+^*], we overexpressed Sup35NM-RFP in [*PIN^+^*] cells with endogenously tagged GFP proteins [91]. As seen previously [96], we found that the molecular chaperones Hsp104, Sis1, and Ssa1 involved in [*PSI^+^*] propagation via their involvement in prion shearing [72], [97]–[104], colocalized with Sup35 rings and dots perfectly during *de novo* induction of [*PSI^+^*] (Fig. 5A, S8 Fig.). However, Ydj1, a Hsp40 co-chaperone shown to co-immunoprecipitate with [*PSI^+^*] aggregates as a minor component along with Hsp104, Ssb, Sis1, Sse1 [104], [105] did not colocalize with Sup35 rings (Fig. 5A).

We also asked if proteins other than chaperones, previously found to influence the maintenance or induction of [*PSI^+^*] [22], [106], [107], would colocalize with newly appearing Sup35 aggregates. We found that Sup45-GFP perfectly colocalized with newly appearing Sup35NM-RFP aggregates, as well as with established [*PSI^+^*] aggregates (Fig. 5B). Upon testing other candidate proteins (Cyc8, New1, Pin3, Pin4, Tup1, Mod5, Sgt2), we found that none of them displayed any colocalization with Sup35NM-RFP aggregates (S9 Fig.).

### Colocalization of Q/N-rich Pin4C vs. non-Q/N rich Mod5 with newly appearing Sup35 aggregates during [*PSI^+^*] induction in the absence of Rnq1

**Figure 6:**
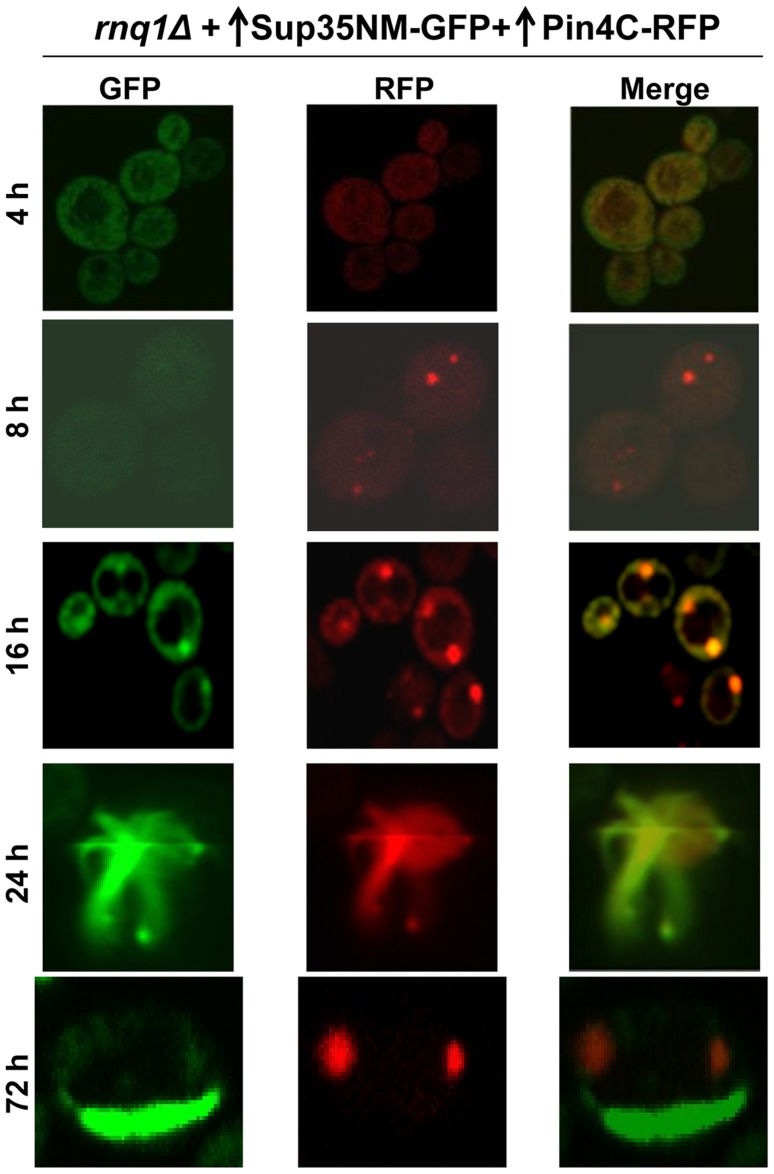
Excess Pin4C temporarily colocalize with Sup35 aggregates during [*PSI^+^*] induction in the absence of Rnq1. Pin4C-RFP (p1708) and Sup35NM-GFP (p1951) were simultaneously overexpressed in 74D-694 *rnq1Δ* cells with 2% Gal for the indicated times. While Sup35NM-GFP early dots and rings (up to 48 h) colocalized with Pin4C-RFP, Pin4C-RFP lost colocalization with Sup35 rings later (72 h) (data in Table 3).

Next, we investigated if high levels of Pin4C (120–668 a.a.), which were previously shown to substitute for [*PIN^+^*] in promoting *de novo* induction of [*PSI^+^*] [22], [66], would overlap Sup35 aggregates during [*PSI^+^*] induction in the absence of Rnq1. We simultaneously overexpressed Pin4C-RFP and Sup35NM-GFP in 74D-694 *rnq1Δ* [*psi* ^-^] cells (Fig. 6, Table 3). Both proteins initially remained diffuse, but by 8 h, tiny Pin4C-RFP fluorescent dots appeared in some of the cells, all of which still had diffuse fluorescence of Sup35NM-GFP. At 16 h, Sup35NM-GFP fluorescent dots appeared and essentially all of these colocalized with Pin4C-RFP near the vacuole. At 24 h, Sup35NM-GFP started to appear as fluorescent rings in addition to dots. In these cells, essentially all Sup35NM-GFP aggregates overlapped Pin4C-RFP. At 48 h, the number of cells with Sup35NM-GFP dots decreased, while cells with rings increased, and all Sup35 aggregates still overlapped Pin4C-RFP. At 72 h, the Sup35NM-GFP dots still colocalized with Pin4C-RFP. However, almost all of the cells that had Sup35NM-GFP rings failed to show Pin4C-RFP rings. Instead, they contained large fluorescence spots of Pin4C-RFP that did not colocalize with Sup35NM-GFP. In control *rnq1Δ* cells that separately overexpressed either Sup35NM-GFP or Pin4C-RFP for 72 h, respectively, Sup35NM-GFP always remained diffuse, while in 50% of the cells (n≈450) Pin4C-RFP formed large fluorescent dots (S10A Fig.). As expected, [*PSI^+^*] appeared *de novo* in *rnq1Δ* cultures with overexpressed Sup35NM-GFP and Pin4C-RFP (S9 Table).

**Table 3:** The data for the fluorescence patterns of Pin4C-RFP and Sup35NM-RFP in *rnq1Δ* cells (refer to Fig. 6).

Time after addition of 2% Gal (h)	Pin4C-RFP fluorescence	Sup35NM-GFP fluorescence	Colocalization of Sup35NM-GFP with Pin4C-RFP
0	Nothing	Nothing	N/A^a^
4	100% diffuse	100% diffuse	N/A
8	6% tiny dots	100% diffuse	N/A
16	9% dots	3% dots	100%
24	11% dots, 1% rings	5% dots, 1% rings	100%
48	13% dots, 5% rings	4% dots, 5% rings	100%
72	16% dots, 0.5% rings	<1% dots, 9% rings	100% dots, ∼5% rings

Pin4C-RFP (p1708) and Sup35NM-GFP (p1951) were co-overexpressed in *rnq1Δ* cells by growth in 2% Gal. After the indicated time of induction, cells with Sup35NM-GFP and/or Pin4C-RFP aggregates were counted and checked for colocalization. Percentages are based on n≈400-600.

a Not Applicable.

To determine the location of Pin4C aggregates relative to the vacuole during Sup35 *de novo* aggregation, we overexpressed Pin4C-RFP in [*pin^-^*] *HSP42-GFP* cells in the presence vs. absence of Sup35NM overexpression (S10B Fig.). In the presence of Sup35NM overexpression, Pin4C-RFP formed one to a few foci, one of which perfectly (in 6.5% of the cells; n∼800) or partially (in 93.5% of the cells) overlapped the Hsp42-GFP dot. In the absence of Sup35NM overexpression, Pin4C-RFP dots never perfectly overlapped the Hsp42-GFP dot; rather one of the Pin4C-RFP aggregates was juxtaposed to, or partially colocalized with the Hsp42-GFP dot. This suggests that Sup35NM overexpression promotes a more frequent closer association of the Pin4C-RFP aggregate and the vacuole-associated Hsp42-GFP protein deposit.

**Figure 7:**
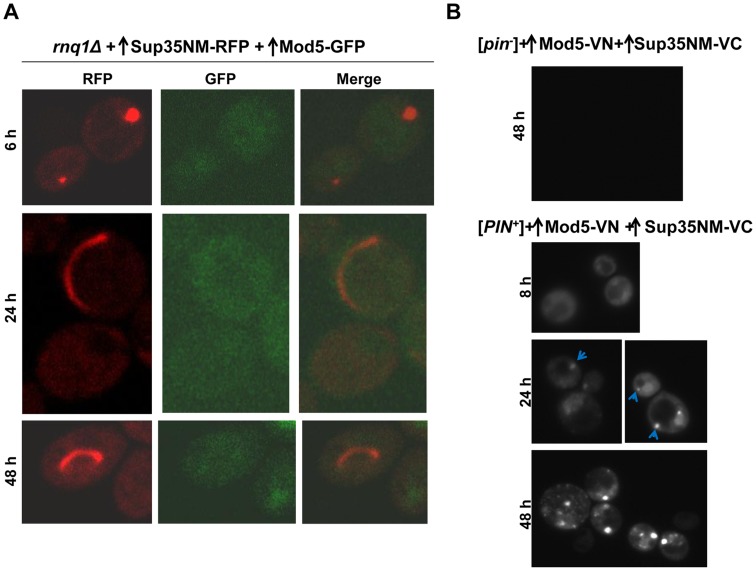
Overexpressed Mod5 does not interact with Sup35 during [*PSI^+^*] induction in the absence of Rnq1. **A.** Mod5 did not form visible aggregates although Sup35 newly appearing aggregates formed. Mod5-GFP (p2061) and Sup35NM-RFP (p2018) were simultaneously overexpressed in 74D-694 *rnq1Δ* cells with 2% Gal for the indicated times. Mod5-GFP remained diffuse although Sup35NM–RFP formed dots and rings (data in Table 4). **B.** Mod5 did not form a close physical interaction with Sup35 during [*PSI^+^*] induction in the absence of [*PIN^+^*]. Mod5-VN (p2170) and Sup35NM-VC (p1892) were simultaneously overexpressed in 74D-694 [*pin^-^*] (top) and [*PIN^+^*] (bottom) cells with 2% Gal for the indicated times. No fluorescence was detected in [*pin^-^*], but Sup35-Mod5 dots appeared in [*PIN^+^*] cells mostly near the vacuole (arrows) following the diffuse fluorescence. (see also S11B Fig.)

When we simultaneously overexpressed the non-Q/N rich prion protein Mod5 tagged with GFP and Sup35NM-RFP in 74D-694 *rnq1Δ* cells, Sup35NM-RFP initially formed dots, and then rings/lines, while Mod5-GFP always remained diffuse (Fig. 7A, Table 4). In control *rnq1Δ* cells that separately overexpressed either Sup35NM-RFP or Mod5-GFP, both proteins remained diffuse (S11A Fig.). Since we could not see fluorescent aggregates of Mod5-GFP, we turned to BiFC to look for an interaction between Mod5 and Sup35NM (Fig. 7B, S11B Fig.). Overexpression of Mod5-VN and Sup35NM-VC did not result in any fluorescence in [*pin^-^*] cells, although [*PSI^+^*] was induced in this culture with a frequency of 0.9% (n∼1000) suggesting that cross-seeding may not be universal for [*PSI^+^*] induction. Curiously, in [*PIN^+^*] cells, overexpression of Mod5-VN and Sup35NM-VC resulted in first diffuse fluorescence and then the formation of a single (near the vacuole) to multiple dots over time in 15% of cells (Fig. 7B). Also, Mod5-VN and Mod5-VC overexpression in [*pin^-^*] cells did not show any fluorescence, but in [*PIN^+^*] cells they showed diffuse fluorescence (S11C Fig.). Possibly, newly appearing Sup35NM aggregates in [*PIN^+^*] cells are attracted to the Mod5 aggregates seen as diffuse fluorescence.

**Table 4:** The data for the fluorescence patterns of Sup35NM-RFP and Mod5-GFP in *rnq1Δ* cells (refer to Fig. 7A).

Time after addition of 2% Gal (h)	Sup35NM-RFP fluorescence	Mod5-GFP fluorescence
6	Dots	Diffuse
24	0.3% rings/lines	Diffuse
48	0.8% rings/lines	Diffuse

Sup35NM-RFP (p2018) and Mod5-GFP (p2061) were co-overexpressed in *rnq1Δ* cells by growth in 2% Gal. After the indicated time of induction, cells with Sup35NM-RFP and Mod5-GFP fluorescence were counted (n≈600).

These findings indicate that during the *de novo* aggregation of overexpressed Sup35 promoted by overexpression of Q/N-rich Pin4C in the absence of Rnq1 [22], [66], Sup35 aggregates initially colocalize with Pin4C aggregates (near the vacuole), but Pin4C falls off the Sup35 rings later. Furthermore, Pin4C-RFP perfectly overlapped Hsp42-GFP only in the presence of Sup35 overexpression. The data is consistent with the cross-seeding of Sup35 aggregation by Pin4C aggregates in the absence of Rnq1. However, although the overexpression of the non-Q/N rich protein Mod5 promotes [*PSI^+^*] induction [27], we could not visualize Mod5-Sup35 direct interaction in the absence of [*PIN^+^*] suggesting that Mod5-promoted *de novo* Sup35 aggregation occurs via a different mechanism.

### The role of Hsp104 in *de novo* [*PSI^+^*] induction

**Figure 8:**
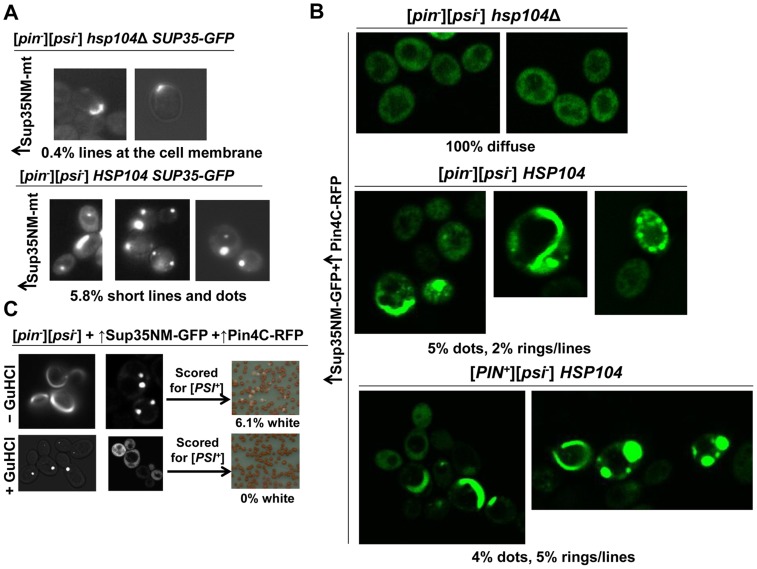
Hsp104 is required for the *de novo* [*PSI^+^*] induction. **A.** Absence of Hsp104 dramatically reduces the frequency of *de novo* Sup35 aggregates. Sup35NM with a short tail of hydrophobic residues (Sup35NM-mt) was overexpressed from p1984 by growing *hsp104Δ* (*GF844*) or [*pin^-^*] *HSP104* (*GF658*) cells with endogenous *SUP35-GFP* in 2% Gal. Sup35-GFP remained mostly diffuse in *hsp104Δ* cells with a few cells with short lines located at the cell membrane, but formed dots and short lines throughout the cytoplasm in [*pin^-^*] *HSP104* cells after 48 h of induction. **B.** Hsp104 is necessary to induce Sup35 newly appearing rings and lines during Pin4C-promoted [*PSI^+^*] induction. Sup35NM-GFP and Pin4C-RFP were respectively co-overexpressed from p1181 and p1708, by growing *hsp104Δ* (L1802, or L1803), *HSP104* [*pin^-^*] (L2910) or *HSP104* [*PIN^+^*] (L1749) cells in 50 µM CuSO_4_ and 2% Gal. In the absence of Hsp104, Sup35NM-GFP failed to form any aggregates in contrast to *HSP104* cells. **C.** Inhibition of Hsp104 does not induce [*PSI^+^*] *de novo*. Sup35NM-GFP and Pin4C-RFP were co-overexpressed respectively from p1181 and p1708, by growing *HSP104* [*pin^-^*] (L2910) cells in 50 µM CuSO_4_, 2% Gal and with or without 10 mM GuHCl. In comparison to dots and ring formation by Sup35NM-GFP in the absence of GuHCl (Fig. 8B), Sup35NM-GFP never formed rings/lines in the presence of GuHCl. 2.8% of cells had dots and 97.2% of cells were diffuse. None of cells grown in the presence of GuHCl induced [*PSI^+^*]. The pictures were taken 72 h after the addition of copper, galactose and GuHCl to the growth media.

The Hsp104 chaperone requirement for the maintenance of [*PSI^+^*] [72] and the colocalization of Hsp104 with Sup35 dots and rings during the induction of [*PSI^+^*] ([96], Fig. 5A) led us to ask if Hsp104 is also required for the *de novo* aggregation of Sup35 during [*PSI^+^*] induction. Since Hsp104 is required for the maintenance of [*PIN* ^+^], and the requirement for [*PIN^+^*] in the *de novo* induction of [*PSI^+^*] can be overcome by overexpressing certain Sup35NM-containing fragments, *e.g.* with a short extension of hydrophobic residues [108], we overexpressed Sup35NM with a short extension of hydrophobic residues (magic tail), previously shown to induce [*PSI^+^*] even in [*pin^-^*] cells [49], [108]. Overexpression of Sup35NM with this magic tail (Sup35NM-mt) in [*pin^-^*] *HSP104* cells, caused endogenous Sup35-GFP molecules to form dots and short lines in 6% of the cells (n≈1200) (Fig. 8A) and this increased to 15% (n≈1400) of the cells in the presence of [*PIN^+^*]. Unlike the Sup35 dots induced in [*PIN* ^+^] cells by 24 h of overexpression of Sup35NM without magic tail, Sup35 dots induced by Sup35NM-mt in [*pin^-^*] *HSP104* or [*PIN^+^*] *HSP104* cells did not always appear near the vacuole. In [*pin^-^*] *hsp104Δ* cells however, no dots were seen and only 0.4% of the cells (n≈1700) formed Sup35-GFP lines. Furthermore, all these lines appeared to be at the cell membrane as opposed to those seen in the cytoplasm in *HSP104* cells.

In an alternative approach to test the role of Hsp104 in [*PSI^+^*] induction, we used Pin4C overexpression to substitute for [*PIN^+^*]. We co-overexpressed Sup35NM-GFP and Pin4C-RFP in [*pin^-^*] *hsp104Δ* cells (Fig. 8B), and Sup35NM-GFP remained diffuse, while in control [*pin^-^*] *HSP104* or [*PIN^+^*] *HSP104* cells, as expected, Sup35NM-GFP formed dots and rings/lines respectively in 7% and 9% of the cells. Also, Pin4C-RFP aggregates were not affected by the presence vs. absence of Hsp104 in [*pin^-^*] cells, but were larger in the presence of [*PIN^+^*] (S12 Fig.). Co-overexpression of Sup35NM-GFP and Pin4C-RFP in [*pin^-^*] *HSP104* cells in the presence of GuHCl, which inhibits Hsp104's ATPase activity [79] resulted in the formation of only Sup35NM-GFP dots in 2.8% of cells, but no rings/lines and failed to induce any [*PSI^+^*] cells (Fig. 8C). Taken together, the striking differences in the level and types of Sup35 aggregate formation in the presence vs. absence of Hsp104 and in the presence vs. absence of GuHCl suggest that Hsp104 is required for the formation of *de novo* Sup35 aggregates, and induction of [*PSI^+^*] *de novo*.

## Discussion

Protein aggregates have been implicated in a wide variety of diseases including Amyotrophic Lateral Sclerosis, Alzheimer's, Parkinson's and prion disease [109], [110]. Interactions between proteins associated with protein misfolding diseases (PMD) are of great interest since molecular cross-talk between disease aggregates of one protein can accelerate the *de novo* appearance of heterologous disease protein aggregates [16], [60], [111]–[115]. Several reports implicate cross-seeding as a mechanism to explain this [17], [18]. Here, our data provide insight into the mechanism of prion induction, which is a model for such heterologous interactions in human diseases. Our data suggest that cross-seeding is not the only mechanism for this cross-talk phenomenon. While our findings that [*PIN^+^*] or Pin4C aggregates physically interact with Sup35 *de novo* aggregates are consistent with the cross-seeding model, the failure of Mod5 to interact with Sup35 suggests the existence of another mechanism, possibly via hindering the chaperone network.

**Figure 9:**
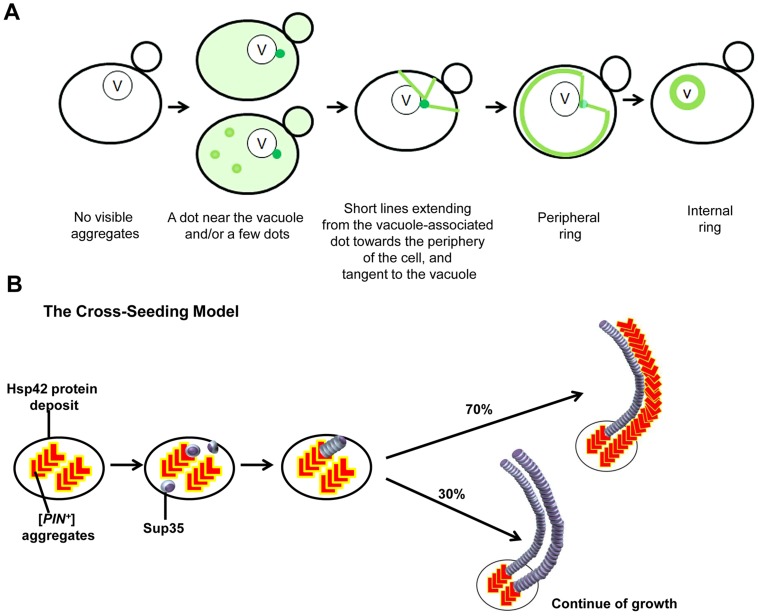
Models we propose to explain *de novo* Sup35 aggregation. **A.** The stages of the induced appearance of [*PSI^+^*] *de novo*. Induction of [*PSI^+^*] by overexpression of fluorescently-labeled Sup35 in [*PIN^+^*][*psi^-^*] cells displays early Sup35 fluorescent foci ranging from one to a few dots, one of which is located near the vacuole (shown as darker green). At this perivacuolar Hsp42 protein deposit site, preexisting heterologous aggregates such as [*PIN^+^*] or Pin4C cross-seed the *de novo* aggregation of Sup35 (see panel B). Later, short lines appear to emanate from this dot towards the periphery of the cell while dots away from the vacuole disappear. Peripheral rings appear next, followed by perivacuolar internal rings. **B.** The cross-seeding model to explain how heterologous aggregates facilitate *de novo* Sup35 aggregation. Preexisting [*PIN^+^*]/Pin4C aggregates (shown in red ‘L’ shape) located at the Hsp42 protein deposit near the vacuole physically interact with soluble misfolded Sup35 proteins (shown as purple marbles). This interaction causes Sup35 to form oligomers. Then, Sup35 grows in length in a homotypic manner. Based on our data, we propose that [*PIN^+^*]/Pin4C aggregates can also grow in length laterally guided by Sup35 fibrils. Over time, laterally growing [*PIN^+^*]/Pin4C aggregates are less frequent. For simplicity, only two fibrils are depicted.

We stimulated the *de novo* generation of a prion in yeast by overexpressing fluorescent derivatives of the prion protein and then monitored its aggregation status. Based on our findings (Fig. 1–3, 6, S1–2 Fig.), we propose a model to explain the pathway followed by Sup35 during its *de novo* conversion into mature [*PSI^+^*] (Fig. 9A). Bimolecular complementation [88], [89] using BiFC-tagged Sup35NM (Fig. 1C) showed that Sup35NM molecules do not associate throughout the cytoplasm, but first interact at specific sites in the cell. This means that the *de novo* aggregation of Sup35 first occurs at these sites rather than being brought to them as pre-existing aggregates. This may be true of other prion-like aggregating proteins as well.

How could prion-like proteins first interact within discrete inclusions? We speculate that upon initial expression, Sup35 is soluble, but upon overexpression, some molecules of the intrinsically unstructured Sup35NM misfold. We suggest that this misfolded protein could be captured by quality control compartments (QCCs) as inclusions, *i.e.* recently discovered Q-bodies [116], where misfolded proteins accumulate *en route* to degradation. The high local concentration of Sup35 at these sites increases the likelihood of prion induction. Furthermore, [*PIN* ^+^] prion aggregates, which are required for [*PSI^+^*] induction [21], [22], [51], are also located at these sites, where they could facilitate nucleation for Sup35 [53], [87], [117], [118] to polymerize Sup35 into amyloid. In Pin4C-promoted [*PSI^+^*] induction, Pin4C appears to take over the role of [*PIN^+^*] aggregates. This suggests that the co-existence of misfolded protein with heterologous amyloid in inclusions accelerates *de novo* conversion of the misfolded protein into amyloid.

However, not all of these inclusions give rise to larger Sup35 fibrils. Rather, Sup35 aggregates away from the perivacuolar site disappear, and Sup35 lines and rings emanate only from the single inclusion in the cell near the vacuole (Fig. 1–3,6). This perivacuolar inclusion also differs from the other inclusions because it alone colocalizes with Hsp42. Furthermore, the finding of only one fluorescent dot in [*PIN^+^*] cells with overexpressed BiFC-tagged Sup35NM and Rnq1 (S6 Fig.) suggests that Sup35 and Rnq1 interact only at the perivacuolar site. It is unclear what happens to the other inclusions, they could be solubilized, degraded or join the remaining perivacuolar aggregate. This suggests that the fibrillar growth of *de novo* aggregates requires site-specific chaperones.

Curiously, heterologous aggregates are not only involved in the initial cross-seeding, but continue to be associated with some newly seeded heterologous fibrils. This is surprising since once nucleated by [*PIN^+^*]/Pin4C amyloid, Sup35 polymerization should continue without the need for Rnq1/Pin4C seed. Indeed, due to the higher efficiency of homotypic polymerization [55], [118], [*PIN^+^*]/Pin4C aggregates are not expected to incorporate into the growing Sup35 fibrils. Paradoxically, Rnq1 frequently (Pin4C always) are found to overlap newly appearing Sup35 rings (Fig. 4, 6). We propose that although the initial step in nucleation is heterotypical, [*PIN^+^*]/Pin4C aggregates might also grow in length homotypically in close proximity to the Sup35 aggregates via a lateral interaction, possibly with the help of chaperones that are associated with the aggregates. Indeed, our findings that all Rnq1/Pin4C rings colocalized with Sup35 rings (S4 Table); and that Rnq1/Pin4C formed mesh/ring-like aggregates only in the presence of Sup35 overexpression (Fig. 4D–E, S6 Fig.) support this hypothesis, which predicts that Sup35 rings template the continued growth of Rnq1/Pin4C into rings and not *vice versa* (see Fig. 9B).

Mature [*PSI^+^*] dots do not associate with [*PIN^+^*] in the same manner as newly induced [*PSI^+^*] aggregates do. Indeed, although Rnq1 was found to co-immunoprecipitate with newly appearing Sup35 aggregates [63], [119], it was not detected in purified [*PSI^+^*] aggregates [104]. Here, we show the formation of fluorescent dots and mesh-like aggregates by co-overexpressed Rnq1-VC and Sup35NM-VN during [*PSI^+^*] induction, and the inability of Rnq1-VC and Sup35NM-VN to cause fluorescence in established [*PSI^+^*], suggesting that Rnq1 and Sup35 are in close proximity during [*PSI^+^*] induction, but not so close in established [*PSI^+^*] (S6 Fig., S8 Table).

Mature and newly induced Sup35 aggregates also differ in their amyloid characteristics as our data showed that all of the mature [*PSI^+^*] aggregates were stained *in situ* with amyloid-binding dye, thioflavin T, while only 30% and 60% of respectively, newly appearing Sup35 dots and rings were stained with ThT (Fig. 2). This could be either because cross-talk between heterologous amyloid aggregates may not always convert the prion-like protein into amyloid vs. amorphous aggregates, or interference of other proteins attracted to aggregates *in situ* may cause false negative results. We favor the latter possibility as most viable Sup35 ring-bearing cells give rise to [*PSI^+^*] progeny [84], [85]. However, it is possible that dead cells have non-amyloid Sup35 rings. Alternatively, it is also possible that newly appearing Sup35 aggregates that are stained with Thioflavin T harbor Rnq1 amyloid, leading to the ThT staining, while those Sup35 aggregates that are not stained do not harbor Rnq1 amyloid, leading to the failure of ThT staining. This would imply that the Sup35 molecules in the dot and ring structures are not amyloid.

While colocalization of a protein with heterologous aggregates is consistent with cross-seeding, it is not proof of cross-seeding. Indeed, the strict and permanent colocalization of Sup45 with Sup35 newly appearing aggregates and with mature [*PSI^+^*] aggregates (Fig. 5B) suggests that Sup45-GFP simply decorates all Sup35 aggregates. However, the considerable and transient colocalization of respectively, Rnq1 and Pin4C with Sup35 newly appearing aggregates, but only partial and no colocalization of respectively, Rnq1 and Pin4C [66] with mature [*PSI^+^*] aggregates supports the idea that as opposed to Sup45, Rnq1 and Pin4C actually cross-seed Sup35 *de novo* aggregates. It is noteworthy that Sup45 is not required for [*PSI^+^*] induction [86], but [*PIN^+^*], or one of its substitutes, Pin4C is.

Also, the colocalization of the molecular chaperone Hsp104 with all Sup35 aggregates (Fig. 5A, [120]) suggests that Hsp104 decorates rather than cross-seeds Sup35. Such decoration could enable Hsp104 to perform its known shearing activity of [*PSI^+^*] aggregates that is required for [*PSI^+^*] propagation [72], [80]–[82]. We showed that Hsp104 itself is also required for *de novo* induction of [*PSI^+^*] by finding inhibition of the *de novo* aggregation of Sup35 in [*pin^-^*] cells lacking Hsp104 (Fig. 8A,B) and inhibition of *de novo* induction of [*PSI^+^*] in [*PIN^+^*] cells in the presence of GuHCl (Fig. 8C), which inhibits Hsp104's activity [79]. This is consistent with a previous report that overexpression of Hsp104 enhances prion appearance [121].

Although cross-seeding is generally thought to explain the cross-talk that enables amyloid aggregates to promote conversion of heterologous prion-like protein to amyloid, our data suggest that another mechanism is also involved. Indeed, we showed that in the absence of [*PIN^+^*], overexpression of the non-Q/N rich prion protein Mod5 enhances [*PSI^+^*] formation *without* direct physical interaction with Sup35 (Fig. 7B, S11B Fig.). Possibly, instead of cross-seeding which requires a physical interaction, excessive amounts of misfolded Mod5 proteins in [*pin^-^*] cells sequester chaperones away from newly forming Sup35 aggregates in the cell, and thus allow them to mature into a prion. Since Mod5-promoted [*PSI^+^*] induction is rare compared to [*PIN^+^*]-promoted induction, cross-seeding appears to be more efficient than the other mechanism in promoting *de novo* prion formation.

[*PIN^+^*] also appears to influence *de novo* aggregation of Mod5. Bimolecular complementation using BiFC-tagged Mod5 (S11C Fig.) showed that Mod5 prion protein interacts with itself, resulting in diffuse fluorescence only in [*PIN^+^*], but not in [*pin^-^*] cells. Curiously, an interaction between Sup35NM-VC and Mod5-VN is also seen as diffuse fluorescence, and again only in [*PIN^+^*] cells (Fig. 7B). Possibly, the Mod5 *diffuse* aggregates present in [*PIN^+^*] cells attract and interact with Sup35NM aggregates.

The data presented here aid our understanding of how prion formation occurs in yeast, and provide clues to the molecular mechanisms underlying many human aggregating neurodegenerative diseases, particularly these arising more frequently in people with preexisting neurodegenerative disease.

## Methods

### Strains and plasmids

Yeast plasmids and strains used in this study are listed in Tables 5 and 6, respectively. All [*PIN^+^*] cells used in the study were high [*PIN^+^*] [34]. GF657, GF658 and GF844 are, respectively, [*PSI^+^*][*PIN^+^*] *HSP104*, [*psi^-^*][*pin* ^-^] *HSP104* and [*psi^-^*][*pin* ^-^] *hsp104Δ* versions of 74-D694 with endogenous *SUP35* replaced with *SUP35-GFP* (kindly supplied as SY80, SY84, and SY97 by T. R. Serio, U. Arizona) [75]. L3107 and L2903 were obtained independently by curing GF657 of [*PSI^+^*] by overexpressing Pin4C [66]. GF852 and GF855, respectively, are [*psi^-^*][*PIN* ^+^] and [*psi^-^*][*pin* ^-^] versions of 74-D694 with *RNQ1-CFP* under its own promoter integrated into the genomic *TRP1* (kindly supplied as 645 and 651 by L. Li, Northwestern U.). GF647 was constructed by replacing chromosomal *RNQ1* with *KanMX4* in 74D-694 [122]. All other yeast strains used in the studies of colocalization with Sup35 are from the BY4741 GFP library strain (Life Technologies, CA) harboring the gene of interest tagged endogenously with GFP [91]. To obtain [*pin^-^*] versions of these cells, they were grown on YPD plates with 5 mM GuHCl for three passes [50].

**Table 5:** Plasmids used in this study.

Plasmids	Description	Reference
p742	pEMBL-YEX (*URA3*)	[49]
p743	pEMBL-*SUP35* (*URA3*)	[49]
p1156	pEMBL-*SUP35NM*-mt (*URA3*)	[49]
p1181	pRS413-*CUP1-NM-GFP* (*HIS3*)	[62], [84]
p1678	pRS416-*GAL1-SUP35-RFP* (*URA3*)	[85]
p1708	pHR81-*GAL1-PIN4C-RFP* (*URA3*)	[66]
p1728	pRS416-*GAL1-RNQ1-YFP* (*URA3*)	[122]
p1730	pRS413-*RNQ1-RNQ1-GFP* (*HIS3*)	[127]
p1752	pRS416-*GAL1-YFP* (*URA3*)	[122]-[124]
p1753	pRS426-*GAL1-SUP35NM-YFP* (*URA3*)	[87], [96], [125], [126]
p1892	pRS414-*GAL1-SUP35NM-VC155* (*TRP1*)	This study and [88]
p1893	pRS413-*GAL1-SUP35NM-VN173* (*HIS3*)	This study and [88]
p1893-2	pRS415-*GAL1-SUP35NM-VN173* (*LEU2*)	This study
p1894	pRS414-*GAL1-RNQ1-VC155* (*TRP1*)	This study
p1951	pRS413-*GAL1-SUP35NM-GFP* (*HIS3*)	This study
p1984	pYES4-*GAL1-SUP35NM-mt* (*URA3*)	This study
p2017	pRS416-*GAL1-SUP35NM-RFP* (*URA3*)	This study
p2018	pRS415-*GAL1-SUP35NM-RFP* (*LEU2*)	This study
p2061	pRS426-*MOD5-GFP* (*URA3*)	[27]
p2036	pRS426*-GAL1-SUP35NM* (*URA3*)	This study
p2170	pRS413-*GAL1-MOD5-VN173* (*HIS3*)	This study
p2171	pRS414-*GAL1-MOD5-VC155* (*TRP1*)	This study

**Table 6:** Yeast strains used in this study.

Strains	Description	Reference
74D-694	MAT*a ade1-14 ura3-52 leu2-3,112 trp1-289 his3-200*	[33]
L1749	74D-694 [*psi^-^*][*PIN^+^*]	[33]
L1802/L1803	74D-694 [*psi^-^*] [*pin^-^*] *hsp104Δ::LEU2*	[50], [72]
L2910	74D-694 [*psi^-^*] [*pin^-^*]	[33]
GF844	74D-694 Mat*α SUP35::N(GF) 3sGFP (GS)3MC hsp104Δ::LEU2* [*psi^-^*] [*pin^-^*]	[75]
L3107/L2903	74D-694 Mat*α SUP35::N(GF) 3sGFP (GS)3MC* [*psi^-^*] [*PIN^+^*]	This study
GF658	74D-694 Mat*α SUP35::N(GF) 3sGFP (GS)3MC* [*psi^-^*] [*pin^-^*]	[75]
GF657	74D-694 Mat*α SUP35::N(GF) 3sGFP (GS)3MC* [*PSI^+^*] [*PIN^+^*]	[75]
GF852	74D-694, [*PIN^+^*] *pRNQ1- RNQ1-CFP::TRP1*	A gift from Liming Li, NWU
GF855	74D-694, [*pin-*] *pRNQ1- RNQ1-CFP::TRP1*	A gift from Liming Li, NWU
BY4741	MAT*a his3Δ1 leu2Δ0 met15Δ0 ura3Δ0*	[91]
Library BY4741 strains	[*PIN^+^*] or [*pin^-^*] versions of BY4741 *YFG* a *::GFP-HIS3MX6*	[91]
GF647	74D-694 *rnq1Δ::KanMX4*	[122]

a *YFG*: Your Favorite Gene.

All overexpression plasmids in this study were driven by the *GAL1* promoter unless otherwise stated. p1951 (Sup35NM-GFP) was constructed by inserting the *SUP35NM BamHI-NotI* fragment, and the *GFP NotI*-*SacI* fragment in-frame into the pRS413*GAL1* plasmid backbone. p1893 (Sup35NM-VN) was constructed by replacing the *GFP NotI*-*SacI* fragment in p1951 with the *VN173 NotI*-*SacI* fragment PCR-amplified from pFA6a*-HIS3MX6-pGAL1-VN173* [88]. p1893-2 was constructed by moving the *GAL1-SUP35NM-VN XhoI-SacI* fragment in p1893 to the pRS415 backbone. p2170 was constructed by replacing the *SUP35NM BamHI-NotI* fragment in p1893 with the *MOD5 BamHI-NotI* fragment PCR-amplified from the genome. p2171 was constructed by replacing the *SUP35NM BamHI-NotI* fragment in p1892 with the *MOD5 BamHI-NotI* fragment PCR-amplified from the genome. p1892 (Sup35NM-VC) was constructed by (1) replacing the *GFP NotI*-*SacI* fragment in p1951 with the *VC155 NotI*-*SacI* PCR-amplified from *pFA6a-HIS3MX6-pGAL1-VC155* [88], and (2) moving this *GAL1-SUP35NM-VC155 XhoI-SacI* fragment into the *XhoI-SacI* sites of pRS414. p1894 was constructed by replacing the *SUP35NM BamHI-NotI* fragment in p1892 with the *RNQ1 BamHI-NotI* fragment PCR-amplified from the genome. p1984 was constructed by cloning the *XmaI-Sal1* fragment of p1156 into p742. p2036 was constructed by replacing the *YFP SpeI*-*XhoI* fragment in p1752 with the *NM(TAA) SpeI*-*XhoI* fragment PCR-amplified from the genome [122]–[124]. p1753 was described previously [87], [96], [125], [126]. p2017 and p2018 vectors (Sup35NM-RFP) with *URA3* or *LEU2* markers, respectively were constructed in a two-step cloning: (1) The *GFP NotI*-*SacI* fragment in p1951 was replaced with the *RFP NotI*-*SacI* fragment amplified from p1708. (2) Then, this *GAL1-NM-RFP XhoI*-*SacI* fragment was cloned into pRS416 (*URA3*) and pRS415 (*LEU2*) backbones, respectively. All other plasmids listed in Table 5 were described previously [49], [62], [66], [85], [122], [127].

### Cultivation procedures

Yeast strains were cultivated using standard media and growth conditions [128]. Rich media contained 2% dextrose (YPD). Synthetic complete media contained all amino acids except for those used for selection and 2% dextrose (SD, 2% Dex) or 2% galactose (2% Gal). Synthetic liquid media contained amino acids lacking the selective ones and 2% raffinose plus (SRGal) or minus 2% Gal (SRaf).

### Induction of [*PSI^+^*] *de novo*

Yeast cells with Sup35 overexpression plasmids were grown in synthetic liquid selection media (SRaf) overnight. Unless otherwise stated, 2% Gal was added to the culture (OD∼0.5) to induce *de novo* Sup35 aggregation. In time course experiments where time exceeded 48 h, cultures were diluted back to OD∼0.5 in a fresh growth media to keep cells in exponential phase.

### Staining of mature [*PSI^+^*] dots

After *de novo* [*PSI^+^*] was induced in [*psi^-^*][*PIN^+^*] cells, they were grown on synthetic dropout media with 2% dextrose (SD) for many generations to maintain [*PSI^+^*] (∼8 days). Then, they were grown in 0.05% Gal for 3-4 h to allow Sup35NM-YFP to decorate existing [*PSI^+^*] aggregates.

### Color and plate assay for [*PSI^+^*] induction

[*PSI^+^*] induced *de novo* was scored in yeast as described previously [33], [49], [72], [129], [130]. [*PSI^+^*] but not [*psi^-^*] causes read-through of the nonsense mutation, *ade1-14*. The *ade1-14* mutation causes the accumulation of a red by-product in the adenine biosynthesis pathway, so [*psi^-^*] *ade1-14* cells are red. In [*PSI^+^*] cells, when nonsense mutations are suppressed, *ade1-14* cells become pink or white in rich media (YPD). In addition, the read-through of the *ade1-14* allele in [*PSI^+^*] allows them to grow in media lacking adenine (SD-Ade), while [*psi^-^*] cells cannot grow on this medium [49]. To confirm that Ade^+^ cells are of [*PSI^+^*] rather than suppressor mutants, they were mated with a tester strain ([*psi^-^*] *SUP35-GFP*, GF658) to look for multiple fluorescent dots formed by endogenous Sup35-GFP in only [*PSI^+^*] cells.

### FM4-64 and Thioflavin T staining

To locate the vacuole, cells were stained with FM4-64 as described previously [90]. Yeast cells were stained with Thioflavin T according to a protocol adapted from ref. [131] with the addition of two extra washes in PMST [0.1M KPO_4_ (pH 7.5), 1 mM MgCl_2_, 1 M Sorbitol, 0.1% Tween 20].

### Visualization of aggregates and colocalization studies

Aggregates formed in cells by fluorescently labeled proteins were examined with a Nikon Eclipse E600 fluorescent microscope (100X oil immersion) and/or an Olympus FV1000 confocal microscope (60X oil immersion, with 1.6 magnifier). Colocalization was visualized by the confocal microscope using the channels of interest and by moving the focal plane up and down. Z-stacks were analyzed to confirm colocalization of proteins with 8-12 layers, with 0.5-1 µm increments. In dual color RFP/GFP studies, the RFP channel was always examined first to prevent visualization of activated GFP in the RFP channel.

### Detection of protein levels

Upon the induction of overexpression of proteins tagged with VN or VC with galactose, cells were lysed as described previously [132]. Equal amounts of total proteins in precleared lysates were analyzed by Western blot using previously described antibodies [27], [63], [66], [104].

## Supporting Information

S1 Fig Visualization of induced *de novo*Sup35 aggregates in cells with endogenous *SUP35-GFP* **. A.** In [*PIN^+^*] cells, similar to Sup35NM (Fig. 1B), high levels of untagged full length Sup35 (p743) induced endogenous Sup35-GFP to form dots earlier than rings during the induction of [*PSI^+^*] (n≈550). **B.** In [*pin^-^*] control cells with endogenous *SUP35-GFP* (GF658), untagged Sup35NM was overexpressed from p2036 in 2% Gal, but there was no aggregation.
(PDF)

S2 Fig Sup35 aggregates initially appear near the vacuole, and extend to the periphery thereafter. Sup35NM-RFP was overexpressed (p2018) in [*PIN^+^*] cells with endogenous *VPH1-GFP* by growth in 2% Gal.
(PDF)

S3 Fig Failure of some Sup35 newly appearing aggregates to colocalize with Rnq1-GFP. **A.** Failure of colocalization in some [*PIN^+^*] cells. Sup35-RFP was overexpressed (p1678) in 2% Gal for 24 h in [*PIN^+^*] cells with p1730 expressing Rnq1-GFP from its own promoter. Rnq1-GFP remained diffuse (top), or as dots (bottom) in, respectively, 20% and 10% of cells with Sup35-RFP rings/lines (see S3 Table). **B.** Failure of colocalization in control [*pin^-^*] cells. Sup35-RFP was overexpressed (p1678) in 2% Gal for 24 h in [*pin^-^*] cells with p1730 expressing Rnq1-GFP. Neither of them aggregated in [*pin^-^*]. **C.** Control cells without Rnq1-GFP. Sup35-RFP was overexpressed (p1678) in 2% Gal for 24 h in [*PIN^+^*] cells without p1730 (Rnq1-GFP). Sup35-RFP showed rings, which did not show any fluorescence through the GFP filter.
(PDF)

S4 Fig Control experiments for colocalization of Sup35NM-YFP aggregates with Rnq1-CFP. **A.** Control [*pin^-^*] cells with integrated *RNQ1-CFP* and overexpressed Sup35NM-YFP. Sup35NM-YFP was overexpressed (p1753) in [*pin^-^*] *RNQ1-CFP* integrants. Both Rnq1-CFP and Sup35NM-YFP remained diffuse after 48 h. **B.** Control [*PIN^+^*] cells with integrated Rnq1-CFP and repressed Sup35NM-YFP. [*PIN^+^*] *RNQ1-CFP* integrants with the p1753 plasmid were grown in repressing media (Glucose) to inhibit Sup35NM-YFP overexpression. As expected, Rnq1-CFP formed dots, while Sup35NM-YFP displayed no signals after 48 h. **C.** Control [*PIN^+^*] cells with integrated Rnq1-CFP and overexpressed YFP. [*PIN^+^*] *RNQ1-CFP* integrants with the empty vector p1752 (YFP) were grown in 2% Gal. Rnq1-CFP showed only dots, indicative of [*PIN^+^*], while YFP always remained diffuse.
(PDF)

S5 Fig Visualization of [*PIN^+^*] aggregates decorated by Rnq1-CFP and Sup35 aggregates induced by Sup35NM-YFP overexpression through different focal planes. **A.** Perfect colocalization of Sup35NM-YFP with Rnq1-CFP. [*PIN^+^*] *RNQ1-CFP* integrants were grown in 2% Gal for 48 h to overexpress Sup35NM-YFP (p1753). Z-stacks of 12 optical sections spaced ∼1 µm apart were collected. Rnq1-CFP perfectly overlapped Sup35NM-YFP in all sections. Representative pictures shown are from layers 5 through 8 out of 12 sections. **B.** Partial colocalization of Sup35NM-YFP with Rnq1-CFP. Representative pictures were taken from another group of cells with 8 z-stacks, layers 3 through 5 are shown. Yellow arrows and blue stars indicate colocalized rings and non-colocalized lines, respectively (see S6 Table).
(PDF)

S6 Fig Rnq1 and Sup35 form a close physical interaction during [*PSI^+^*< induction, but do not in established [*PSI^+^*]. Sup35NM-VN (p1893) and Rnq1-VC (p1894) in [*PIN^+^*][*psi^-^*] or [*PIN^+^*][*PSI^+^*] 74D-694 cells were co-overexpressed by growth in 0.2% Gal for the indicated times. (n≈600). Expression levels of Sup35NM-VN and Rnq1-VC (bottom) were detected by respectively, α-Sup35N and α-Rnq1 (a kind gift of S. Lindquist) in [*PIN^+^*][*psi^-^*] (left) or [*PIN^+^*][*PSI^+^*] (right) cells harboring p1893 and p1894, and grown in 0.2% Gal for 48 h. Pgk1 was used as an internal loading control.
(PDF)

S7 Fig Failure of Rnq1-CFP to form aggregates other than dots in control cells. **A.** Visualization of Rnq1-CFP in control [*pin^-^*] cells overexpressing untagged Sup35NM. Sup35NM was overexpressed (p2036) in [*pin^-^*] *RNQ1-CFP* integrants by growth in 2% Gal for 48 h. Rnq1-CFP always displayed diffuse fluorescence. **B.** Visualization of Rnq1-CFP in control [*PIN^+^*] cells without Sup35NM overexpression. [*PIN^+^*] *RNQ1-CFP* integrants lacking p2036 (Sup35NM) were grown in 2% Gal. Rnq1-CFP displayed only fluorescent dots by 48 h in 90% of the cells, while 10% of the cells contained diffuse fluorescence.
(PDF)

S8 Fig Colocalization of newly induced Sup35 aggregates with chaperones. **A.** Sup35 aggregates colocalized with Ssa1 and Sis1 chaperones. [*PIN^+^*] cells with GFP-tagged *SSA1* or *SIS1* endogenously were induced to overexpress Sup35NM-RFP (p2017) for 48 h by growth in 2% Gal. Observed Sup35NM-RFP rings in *SSA1-GFP* cells (5.6%, n≈450) completely colocalized with Ssa1-GFP. Sup35NM-RFP rings (5.5%, n≈400) completely colocalized with Sis1-GFP in *SIS1-GFP* cells. **B.** Hsp104 did not form aggregates in [*pin^-^*] cells. Overexpressed Sup35NM-RFP (p2017) in [*pin^-^*] *HSP104-GFP* cells with 2% Gal for 48 h resulted in no aggregate formation.
(PDF)

S9 Fig Failure of newly induced Sup35 aggregates to colocalize with various proteins. **A.** Q/N-rich proteins. Among the Q/N rich proteins that, when overexpressed, can facilitate overexpression of the Sup35 prion domain to form [*PSI^+^*] are Swi1, Cyc8, New1, Pin3 and Pin4 [22], [50], [52], [66], [133]. Swi1 and Cyc8 were later determined to propagate as, respectively, the [*SWI^+^*] and [*OCT^+^*] prions [23], [25]. Likewise, a fusion of the prion domain of New1 with the essential translation termination domain of Sup35 formed the artificial [*NU^+^*] prion [51]. In contrast, Pin3 (*aka* Lsb2), was shown not to form a prion, but to colocalize transiently with some Sup35 aggregates during Pin3-promoted [*PSI^+^*] induction presumably involving the actin cytoskeleton [133]. Finally, overexpression of the residues (120-668 a.a.) of the Pin4 protein (Pin4C) promotes the *de novo* induction of the [*PSI^+^*] prion [22], and also leads to the loss of preexisting [*PSI^+^*] [66]. [*PIN^+^*] cells with one of the Q/N rich proteins (YFG = *CYC8, NEW1, PIN3, or PIN4*) labeled endogenously with GFP, were induced with 2% Gal to overexpress Sup35NM-RFP (p2017) for 48 h. Cyc8-GFP gave a nuclear diffuse signal while New1-GFP, Pin3-GFP and Pin4-GFP were cytoplasmic and diffuse. However, none of these Q/N rich proteins showed colocalization with the Sup35NM-RFP aggregates observed in ∼7% of the cells (n≈450) during [*PSI^+^*] induction. *YFG: Your Favorite Gene.* **B.** Non-Q/N-rich proteins. [*PIN^+^*] cells with endogenously GFP tagged proteins that influence [*PSI^+^*] induction, Mod5 [27], Sgt2 [106] were induced to overexpress Sup35NM-RFP (p2017) by growth in 2% Gal. Neither protein colocalized with the Sup35NM-RFP aggregates observed in ∼7% of the cells (n≈350). To rule out the possibility that Sup45 and Sup35 are colocalizing because of their common association with ribosomes, we tested a ribosomal protein, Rpl5, as a control (Fig. 5B). As expected, Sup35NM-RFP formed rings in 7.2% of the cells with no sign of colocalization with Rpl5-GFP, which remained all diffuse.
(PDF)

S10 Fig Colocalization of Pin4C-RFP with Sup35NM-GFP and Hsp42-GFP. **A.** Pin4C-RFP does not form rings in the absence of Sup35NM-GFP overexpression, and Sup35NM-GFP does not form aggregates without Pin4C overexpression. 74D-694 *rnq1Δ* cells, which contained either p1708 or 1951, were grown in 2% Gal to separately overexpress respectively, Pin4C-RFP or Sup35NM-GFP for 72 h. Pin4C-RFP formed large fluorescence dots, while Sup35NM-RFP remained diffuse. **B.** Sup35NM overexpression changes the location of Pin4C relative to the Hsp42-GFP dot. Pin4C-RFP was overexpressed from p1708 in [*pin^-^*] *HSP42-GFP* cells in the presence (top) or absence (bottom) of Sup35NM overexpression (from p1893-2).
(PDF)

S11 Fig Control experiments for the relationship between Mod5-GFP and Sup35NM-RFP. **A.** Overexpression of neither Mod5-GFP nor Sup35NM-RFP individually formed aggregates in *rnq1Δ*. 74D-694 *rnq1Δ* cells, which contained either p2061 or 2018, were grown in 2% Gal to separately overexpress Mod5-GFP or Sup35NM-RFP, respectively for 48 h. Both Mod5-GFP and Sup35NM-RFP remained diffuse. **B.** Expression levels of Sup35NM-VC and Mod5-VN after 48 h of induction by 2% Gal were detected by α-Mod5 (kind gift of M.Tanaka, [27]) and α-Sup35N in [*pin^-^*][*psi^-^*] vs. [*PIN^+^*][*psi^-^*] cells (see Fig. 7B). **C.** Mod5 forms tiny aggregates in [*PIN^+^*]. Mod5-VN (from p2170) and Mod5-VC (from p2171) were co-overexpressed in [*pin^-^*] (top) and [*PIN^+^*] (bottom) for 48 h by growth on 2% Gal. In [*pin^-^*], Mod5 BiFC did not show any fluorescence, while in [*PIN^+^*], it showed diffuse fluorescence. Expression levels of Mod5 from BiFC constructs p2170 and p2171 were detected by α-Mod5 in [*pin^-^*][*psi^-^*] vs. [*PIN^+^*][*psi^-^*] cells grown in 2% Gal for 48 h. Note that a lower percentage (7% vs 10%) of SDS-PAGE was used to resolve Mod5-VN and Mod5-VC, which are only 9 kDa apart.
(PDF)

S12 Fig Pin4C aggregates were not affected by the presence vs. absence of *HSP104*, but were larger in the presence of [*PIN^+^*]. Sup35NM-GFP and Pin4C-RFP were respectively co-overexpressed from p1181 and p1708, by growing *hsp104Δ* (L1802, or L1803), *HSP104* [*pin^-^*] (L2910) or *HSP104* [*PIN^+^*] (L1749) cells in 50 µM CuSO_4_ and 2% Gal. Pin4C-RFP aggregates were larger and more numerous in [*PIN^+^*] than [*pin^-^*] cells [134] but were the same in the presence or absence of Hsp104 in [*pin^-^*] cells.
(PDF)

S1 Table Visualization of Sup35NM-GFP aggregation in [*PIN^+^*] cells by growth in 0.2% Gal. Sup35NM-GFP in 74D-694 [*PIN^+^*][*psi^-^*] cells was overexpressed from p1951 by growth in 0.2% Gal.
(PDF)

S2 Table Colocalization data of Rnq1-GFP with Sup35-RFP after 6 h of induction of Sup35-RFP in [*PIN^+^*] cells. After 6 h of induction of Sup35-RFP (p1678) by growth of 74D-694 [*PIN^+^*][*psi^-^*] cells with p1730 expressing Rnq1-GFP from its own promoter in 2% Gal, 242 cells were seen to have Sup35-RFP dots out of 6000 cells counted. Among these 242 cells, 144 also showed Rnq1-GFP dots colocalized with Sup35-RFP, but the other 98 cells had diffuse Rnq1-GFP.
(PDF)

S3 Table Colocalization data of Rnq1-GFP with Sup35-RFP after 24 h of induction of Sup35-RFP in [*PIN^+^*] cells. After 24 h of induction of Sup35-RFP (p1678) by growth of 74D-694 [*PIN^+^*][*psi^-^*] cells with p1730 expressing Rnq1-GFP on its own promoter in 2% Gal, 575 cells were seen to have Sup35-RFP lines/rings out of 4800 cells counted. Among these 575 cells, 401 also showed Rnq1-GFP rings/lines colocalized with Sup35-RFP rings/lines, while 57 had Rnq1-GFP dots and the other 117 had diffuse Rnq1-GFP.
(PDF)

S4 Table Colocalization data of Sup35-RFP with Rnq1-GFP after 24 h of induction of Sup35-RFP in [*PIN^+^*] cells. After 24 h of induction of Sup35-RFP (p1678) by growth of 74D-694 [*PIN^+^*][*psi^-^*] cells with p1730 expressing Rnq1-GFP on its own promoter in 2% Gal, 480 cells were seen to have Rnq1-GFP lines or rings out of 6000 cells counted. Among these 480 cells, 474 also showed Sup35-RFP rings/lines colocalized with Rnq1-GFP, but the other 6 cells had diffuse Sup35-RFP.
(PDF)

S5 Table Colocalization data of Rnq1-CFP with Sup35NM-YFP after 24 h of induction of Sup35NM-YFP in [*PIN^+^*] cells. After 24 h of induction of Sup35NM-YFP (p1753) by growth of 74D-694 [*PIN^+^*][*psi^-^*] *RNQ1-CFP* cells with p1753 in 2% Gal, 63 cells were seen to have Sup35NM-YFP dots, and 7 cells had Sup35NM-YFP rings/lines out of 900 cells counted. Among these 63 dot-bearing cells, 57 also showed Rnq1-CFP dots colocalized with Sup35NM-YFP, while the other 6 cells had diffuse Rnq1-CFP. However, among the 7 Sup35-RFP ring bearing cells, they all had Rnq1-CFP rings colocalized with Sup35-RFP.
(PDF)

S6 Table Colocalization data of Rnq1-CFP with Sup35NM-YFP after 48 h of induction of Sup35NM-YFP in [*PIN^+^*] cells. After 48 h of induction of Sup35NM-YFP (p1753) by growth of 74D-694 [*PIN^+^*][*psi^-^*] *RNQ1-CFP* cells with p1753 in 2% Gal, 216 cells were seen to have Sup35NM-YFP lines/rings out of 2000 cells counted. Among these 216 cells, 162 also showed Rnq1-CFP lines/rings colocalized with Sup35NM-YFP, while 15 harbored Rnq1-CFP dots and the other 39 had diffuse Rnq1-CFP.
(PDF)

S7 Table Colocalization data of Sup35NM-YFP with Rnq1-CFP after 48 h of induction of Sup35NM-YFP in [*PIN^+^*] cells. After 48 h of induction of Sup35NM-YFP (p1753) by growth of 74D-694 [*PIN^+^*][*psi^-^*] *RNQ1-CFP* cells with p1753 in 2% Gal, 375 cells were seen to have Rnq1-CFP lines or rings out of 5000 cells counted. Among these 375 cells, 371 also showed Sup35NM-YFP rings/lines colocalized with Rnq1-CFP, but the other 4 cells had diffuse Sup35NM-YFP.
(PDF)

S8 Table Data for overexpressed Sup35 and Rnq1 from the BiFC constructs Sup35NM-VN and Rnq1-VC, respectively by growing cells in 0.2% Gal^a^. Sup35NM-VN (p1893) and Rnq1-VC (p1894) in 74D-694 [*PIN^+^*][*psi^-^*] or [*pin^-^*][*psi^-^*] cells were co-overexpressed by growth in 0.2% Gal. The control [*pin^-^*] cells showed no fluorescence at any time. BiFC fluorescence was checked by YFP filter. Percentages are based on n≈600.
(PDF)

S9 Table Data of scoring for [*PSI^+^*] in *rnq1Δ* cells in the presence of Sup35NM and Pin4C overexpression. To see if observed Sup35NM-GFP aggregates in the absence of Rnq1 when Pin4C-RFP was overexpressed (p1708) were associated with the appearance of [*PSI^+^*] (Fig. 6), we scored cells grown in 2% Gal for different times for [*PSI^+^*] by color assay and growth on SD-Ade. Interestingly, cells taken after 16 h or less of induction with 2% Gal did not grow on SD-Ade at all, and were red on YPD. Cells induced for 24, 48, 72 h were able to grow on SD-Ade with a frequency of 2%, 2.9%, 5%, respectively. In addition, cells from the very same cultures with 24, 48, and 72 h of induction accumulated white/pink color when spread on YPD with a frequency of 1.5%, 3.2%, and 5.9%, respectively.
(PDF)
