# Supplementary material for: Heterologous Aggregates Promote De Novo Prion Appearance via More than One Mechanism
Source: PLoS Genet. 2015 Jan 8;11(1):e1004814. doi: 10.1371/journal.pgen.1004814 (PMC4287349; doi:10.1371/journal.pgen.1004814)
Supplement: S1 Fig — Visualization of induced de novoSup35 aggregates in cells with endogenous SUP35-GFP . A. In [PIN+] cells, similar to Sup35NM (Fig. 1B), high levels of untagged full length Sup35 (p743) induced endogenous Sup35-GFP to form dots earlier than rings during the induction of [PSI+] (n≈550). B. In [pin-] control cells with endogenous SUP35-GFP (GF658), untagged Sup35NM was overexpressed from p2036 in 2% Gal, but there was no aggregation. (PDF) [file pgen.1004814.s001.pdf]

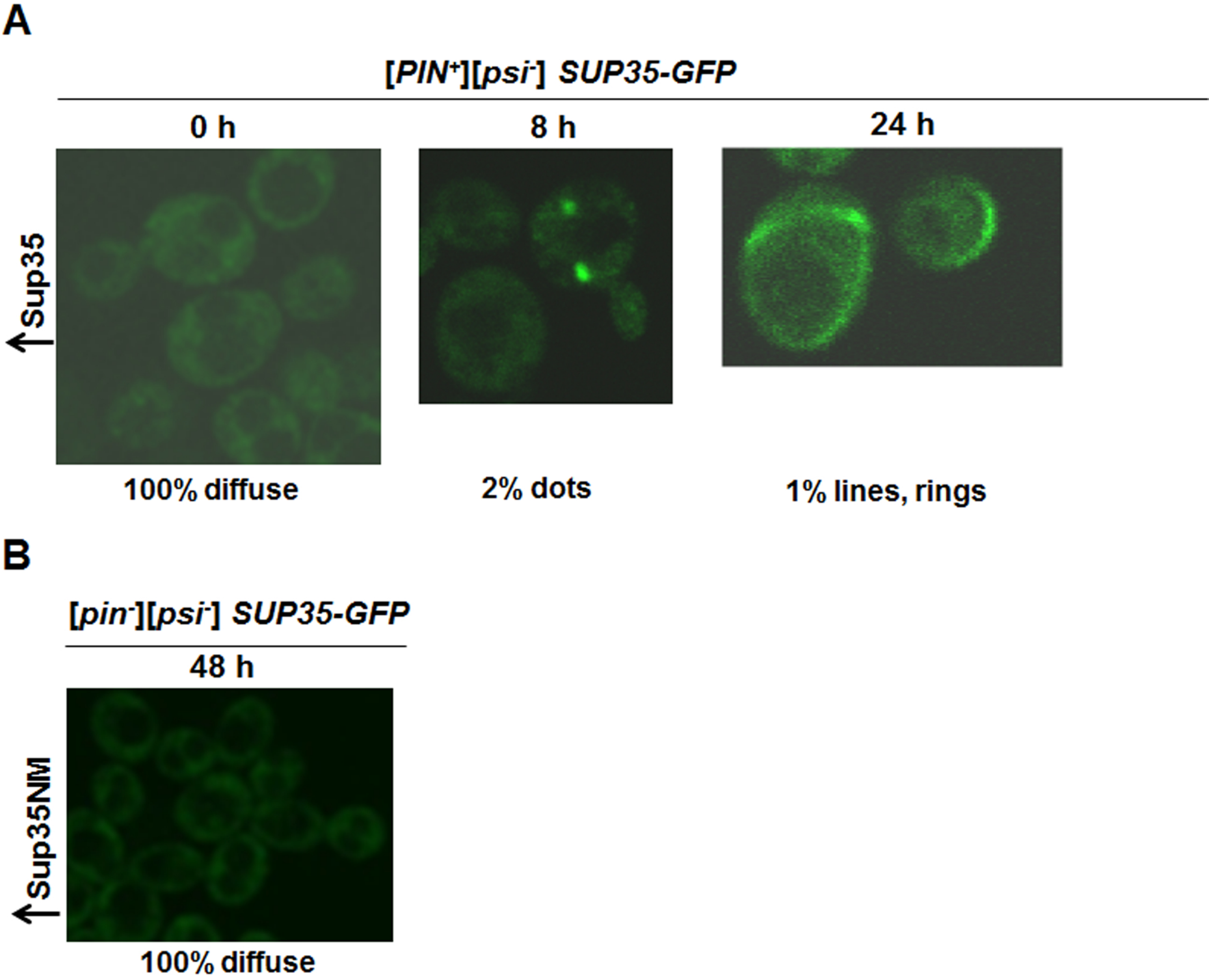
A
[PIN+][psi-] SUP35-GFP
0 h
8 h
24 h
Sup35
100% diffuse
2% dots
1% lines, rings
B
[pin-][psi-] SUP35-GFP
48 h
Sup35NM
100% diffuse
