# Supplementary figures and images for: Heterologous Aggregates Promote De Novo Prion Appearance via More than One Mechanism
Source: PLoS Genet. 2015 Jan 8;11(1):e1004814. doi: 10.1371/journal.pgen.1004814 (PMC4287349; doi:10.1371/journal.pgen.1004814)

**[PIN<sup>+</sup>][psi<sup>-</sup>] VPH1-GFP + ↑Sup35NM-RFP**

**GFP**

**RFP**

**Merge**

**4 h**

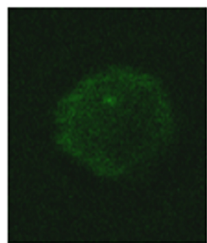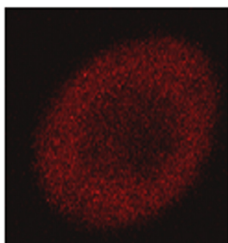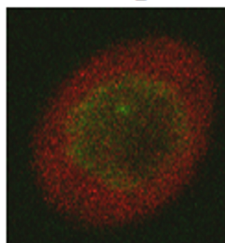

**8 h**

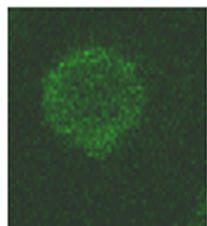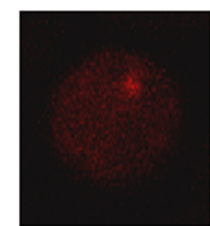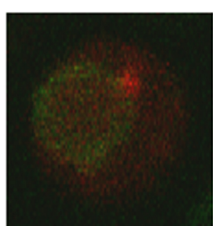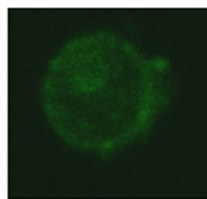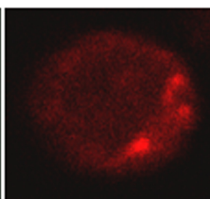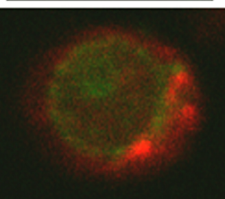

**24 h**

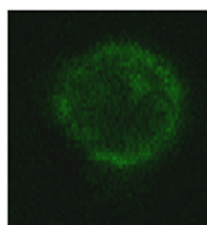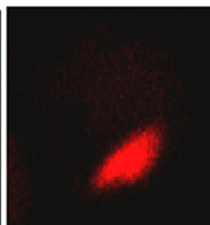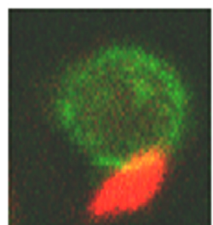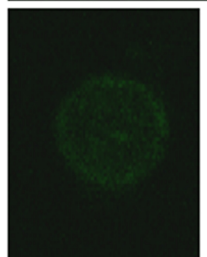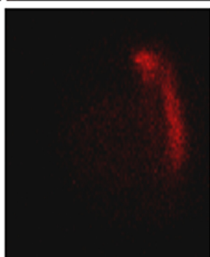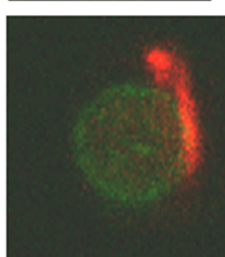

**48 h**

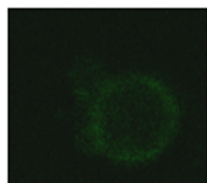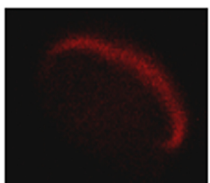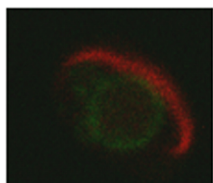

**96 h**

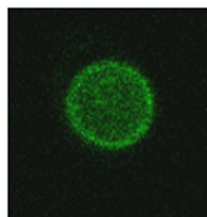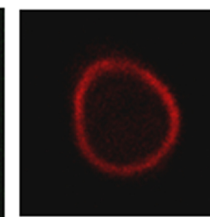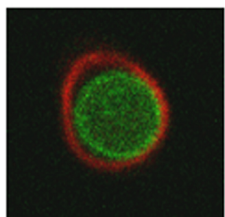

Supplement: S2 Fig — Sup35 aggregates initially appear near the vacuole, and extend to the periphery thereafter. Sup35NM-RFP was overexpressed (p2018) in [PIN+] cells with endogenous VPH1-GFP by growth in 2% Gal. (PDF) [file pgen.1004814.s002.pdf]

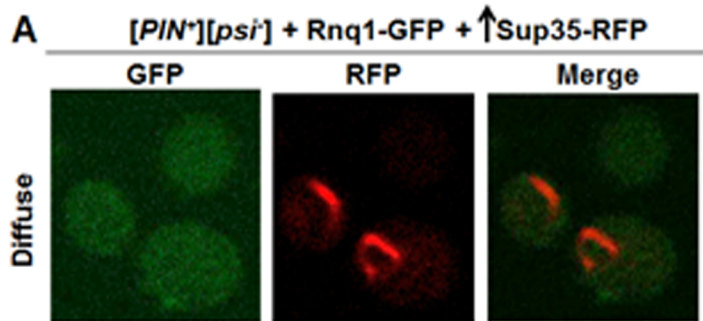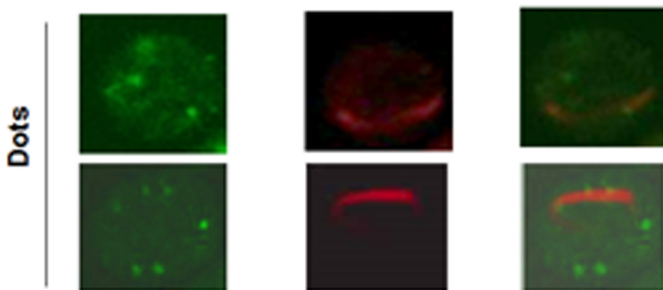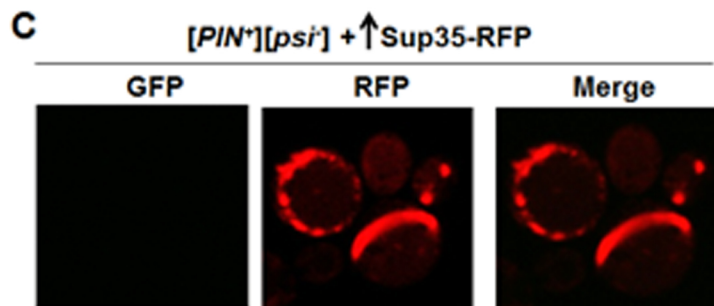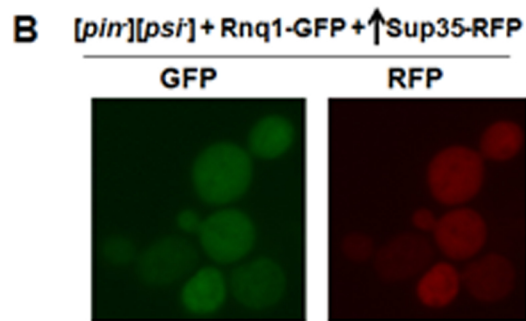

Supplement: S3 Fig — Failure of some Sup35 newly appearing aggregates to colocalize with Rnq1-GFP. A. Failure of colocalization in some [PIN+] cells. Sup35-RFP was overexpressed (p1678) in 2% Gal for 24 h in [PIN+] cells with p1730 expressing Rnq1-GFP from its own promoter. Rnq1-GFP remained diffuse (top), or as dots (bottom) in, respectively, 20% and 10% of cells with Sup35-RFP rings/lines (see S3 Table). B. Failure of colocalization in control [pin-] cells. Sup35-RFP was overexpressed (p1678) in 2% Gal for 24 h in [pin-] cells with p1730 expressing Rnq1-GFP. Neither of them aggregated in [pin-]. C. Control cells without Rnq1-GFP. Sup35-RFP was overexpressed (p1678) in 2% Gal for 24 h in [PIN+] cells without p1730 (Rnq1-GFP). Sup35-RFP showed rings, which did not show any fluorescence through the GFP filter. (PDF) [file pgen.1004814.s003.pdf]

**A**

$[PIN^+][psi^-]$  *RNQ1-CFP* +  $\uparrow$ Sup35NM-YFP

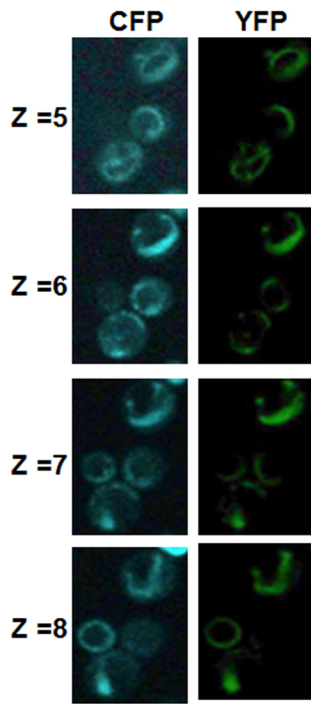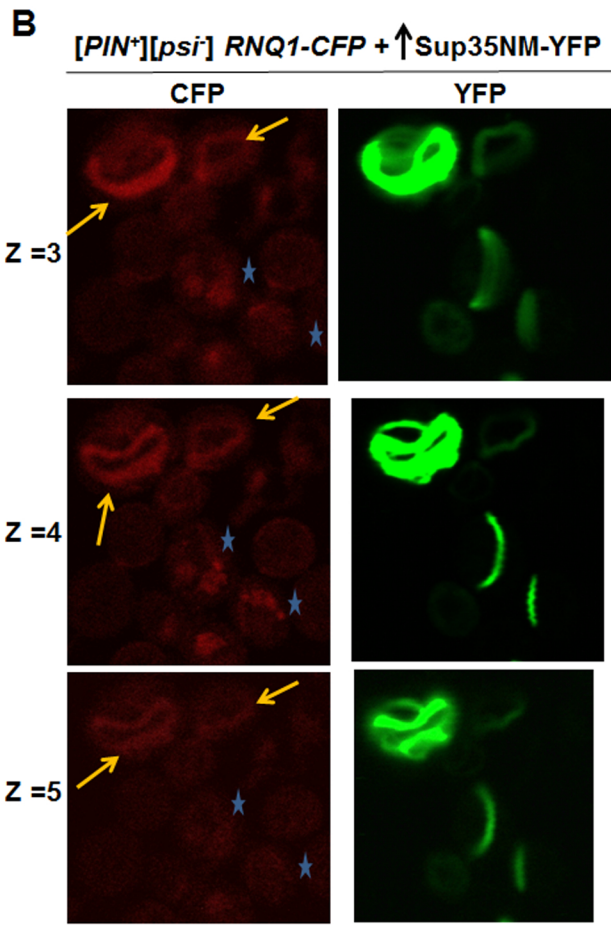

Supplement: S5 Fig — Visualization of [PIN+] aggregates decorated by Rnq1-CFP and Sup35 aggregates induced by Sup35NM-YFP overexpression through different focal planes. A. Perfect colocalization of Sup35NM-YFP with Rnq1-CFP. [PIN+] RNQ1-CFP integrants were grown in 2% Gal for 48 h to overexpress Sup35NM-YFP (p1753). Z-stacks of 12 optical sections spaced ∼1 µm apart were collected. Rnq1-CFP perfectly overlapped Sup35NM-YFP in all sections. Representative pictures shown are from layers 5 through 8 out of 12 sections. B. Partial colocalization of Sup35NM-YFP with Rnq1-CFP. Representative pictures were taken from another group of cells with 8 z-stacks, layers 3 through 5 are shown. Yellow arrows and blue stars indicate colocalized rings and non-colocalized lines, respectively (see S6 Table). (PDF) [file pgen.1004814.s005.pdf]

**A**

***[pin<sup>-</sup>][psi<sup>-</sup>] RNQ1-CFP***

↑ Sup35NM

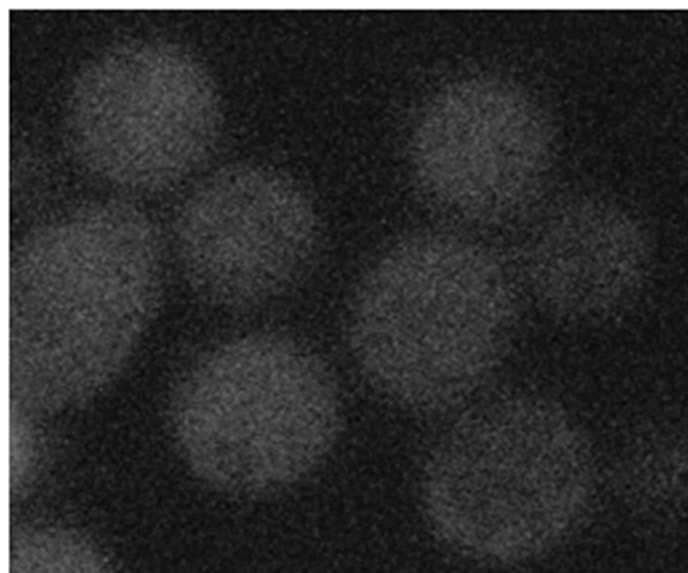

**B**

***[PIN<sup>+</sup>][psi<sup>-</sup>] RNQ1-CFP***

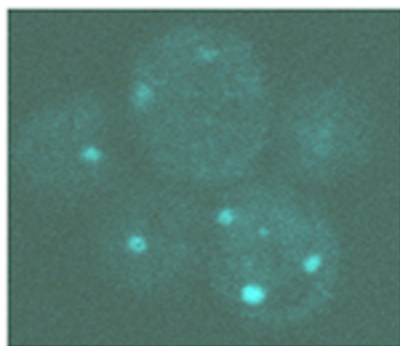

Supplement: S7 Fig — Failure of Rnq1-CFP to form aggregates other than dots in control cells. A. Visualization of Rnq1-CFP in control [pin-] cells overexpressing untagged Sup35NM. Sup35NM was overexpressed (p2036) in [pin-] RNQ1-CFP integrants by growth in 2% Gal for 48 h. Rnq1-CFP always displayed diffuse fluorescence. B. Visualization of Rnq1-CFP in control [PIN+] cells without Sup35NM overexpression. [PIN+] RNQ1-CFP integrants lacking p2036 (Sup35NM) were grown in 2% Gal. Rnq1-CFP displayed only fluorescent dots by 48 h in 90% of the cells, while 10% of the cells contained diffuse fluorescence. (PDF) [file pgen.1004814.s007.pdf]

**A**  $[PIN^+][psi^-]$  YFG-GFP +  $\uparrow$ Sup35NM-RFP

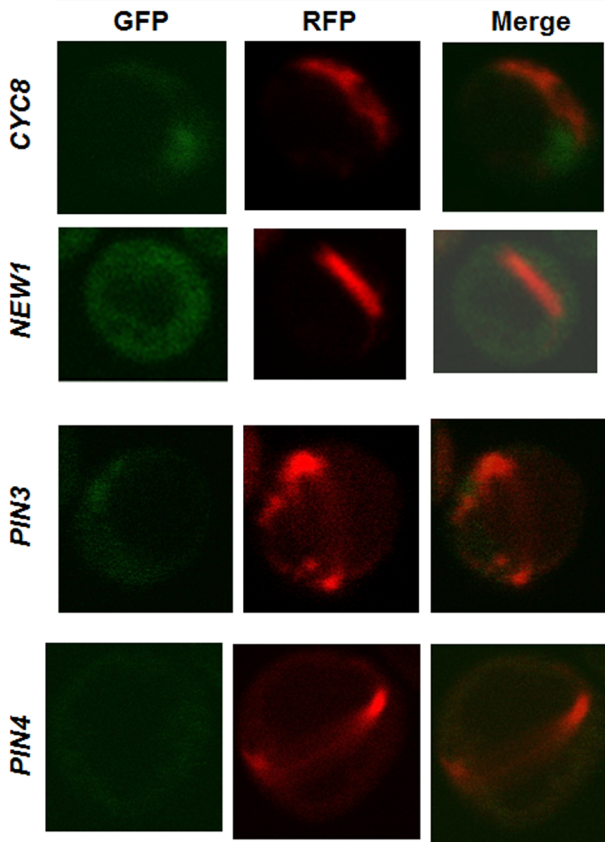

**B**  $[PIN^+][psi^-]$  YFG-GFP +  $\uparrow$ Sup35NM-RFP

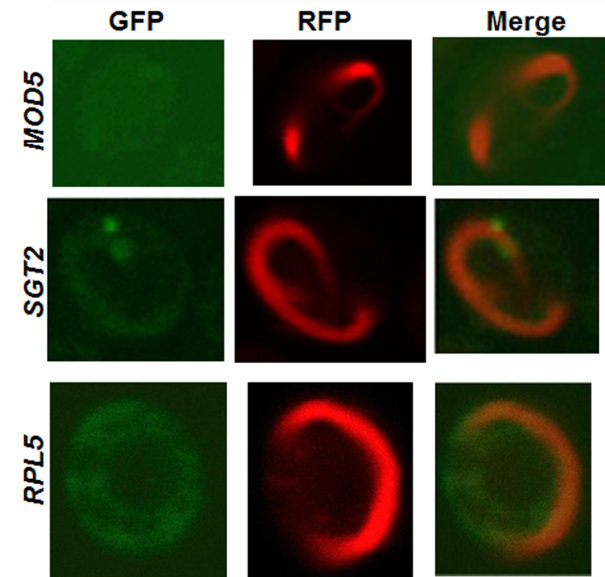

Supplement: S9 Fig — Failure of newly induced Sup35 aggregates to colocalize with various proteins. A. Q/N-rich proteins. Among the Q/N rich proteins that, when overexpressed, can facilitate overexpression of the Sup35 prion domain to form [PSI+] are Swi1, Cyc8, New1, Pin3 and Pin4 [22], [50], [52], [66], [133]. Swi1 and Cyc8 were later determined to propagate as, respectively, the [SWI+] and [OCT+] prions [23], [25]. Likewise, a fusion of the prion domain of New1 with the essential translation termination domain of Sup35 formed the artificial [NU+] prion [51]. In contrast, Pin3 (aka Lsb2), was shown not to form a prion, but to colocalize transiently with some Sup35 aggregates during Pin3-promoted [PSI+] induction presumably involving the actin cytoskeleton [133]. Finally, overexpression of the residues (120-668 a.a.) of the Pin4 protein (Pin4C) promotes the de novo induction of the [PSI+] prion [22], and also leads to the loss of preexisting [PSI+] [66]. [PIN+] cells with one of the Q/N rich proteins (YFG = CYC8, NEW1, PIN3, or PIN4) labeled endogenously with GFP, were induced with 2% Gal to overexpress Sup35NM-RFP (p2017) for 48 h. Cyc8-GFP gave a nuclear diffuse signal while New1-GFP, Pin3-GFP and Pin4-GFP were cytoplasmic and diffuse. However, none of these Q/N rich proteins showed colocalization with the Sup35NM-RFP aggregates observed in ∼7% of the cells (n≈450) during [PSI+] induction. YFG: Your Favorite Gene. B. Non-Q/N-rich proteins. [PIN+] cells with endogenously GFP tagged proteins that influence [PSI+] induction, Mod5 [27], Sgt2 [106] were induced to overexpress Sup35NM-RFP (p2017) by growth in 2% Gal. Neither protein colocalized with the Sup35NM-RFP aggregates observed in ∼7% of the cells (n≈350). To rule out the possibility that Sup45 and Sup35 are colocalizing because of their common association with ribosomes, we tested a ribosomal protein, Rpl5, as a control (Fig. 5B). As expected, Sup35NM-RFP formed rings in 7.2% of the cells with no sign of colocalization with Rpl5-GF [file pgen.1004814.s009.pdf]

**A***rnq1Δ* + ↑ Mod5-GFP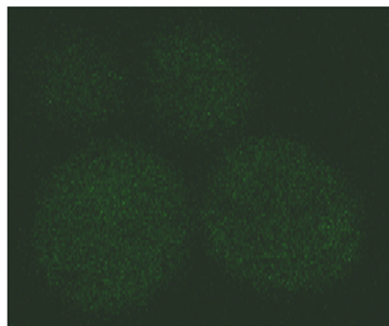*rnq1Δ* + ↑ Sup35NM-RFP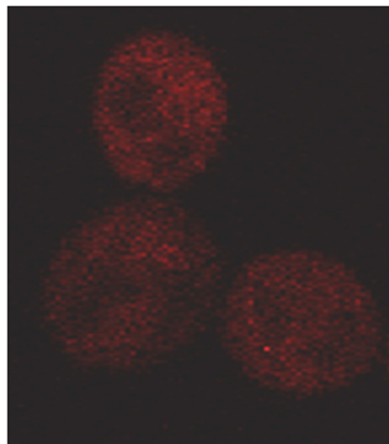**B**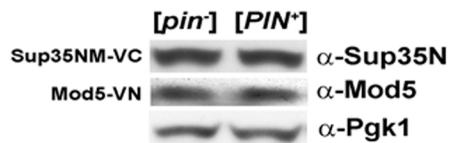**C***[pin-]* + ↑ Mod5-VN + ↑ Mod5-VC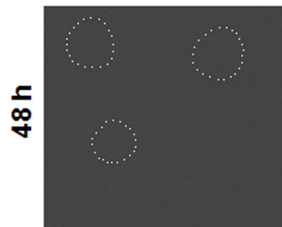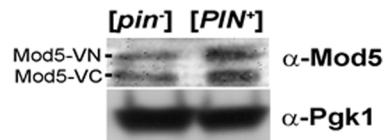*[PIN<sup>+</sup>]* + ↑ Mod5-VN + ↑ Mod5-VC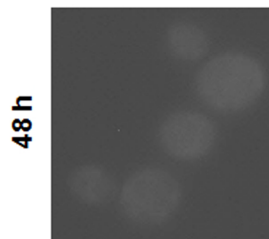

Supplement: S11 Fig — Control experiments for the relationship between Mod5-GFP and Sup35NM-RFP. A. Overexpression of neither Mod5-GFP nor Sup35NM-RFP individually formed aggregates in rnq1Δ. 74D-694 rnq1Δ cells, which contained either p2061 or 2018, were grown in 2% Gal to separately overexpress Mod5-GFP or Sup35NM-RFP, respectively for 48 h. Both Mod5-GFP and Sup35NM-RFP remained diffuse. B. Expression levels of Sup35NM-VC and Mod5-VN after 48 h of induction by 2% Gal were detected by α-Mod5 (kind gift of M.Tanaka, [27]) and α-Sup35N in [pin-][psi-] vs. [PIN+][psi-] cells (see Fig. 7B). C. Mod5 forms tiny aggregates in [PIN+]. Mod5-VN (from p2170) and Mod5-VC (from p2171) were co-overexpressed in [pin-] (top) and [PIN+] (bottom) for 48 h by growth on 2% Gal. In [pin-], Mod5 BiFC did not show any fluorescence, while in [PIN+], it showed diffuse fluorescence. Expression levels of Mod5 from BiFC constructs p2170 and p2171 were detected by α-Mod5 in [pin-][psi-] vs. [PIN+][psi-] cells grown in 2% Gal for 48 h. Note that a lower percentage (7% vs 10%) of SDS-PAGE was used to resolve Mod5-VN and Mod5-VC, which are only 9 kDa apart. (PDF) [file pgen.1004814.s011.pdf]

*[pin<sup>-</sup>][psi<sup>-</sup> hsp104Δ*

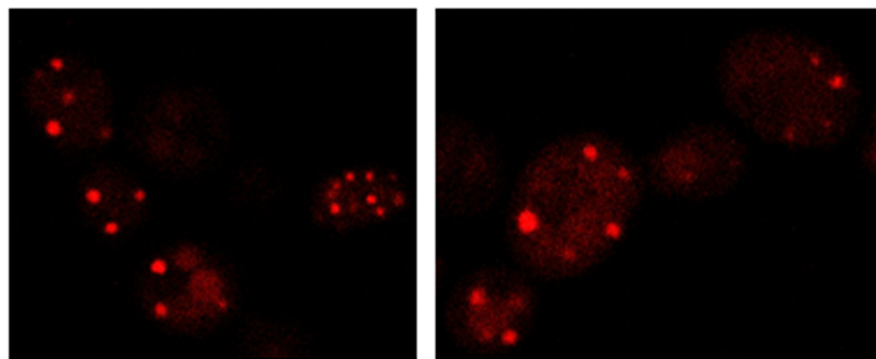

*[pin<sup>-</sup>][psi<sup>-</sup> HSP104*

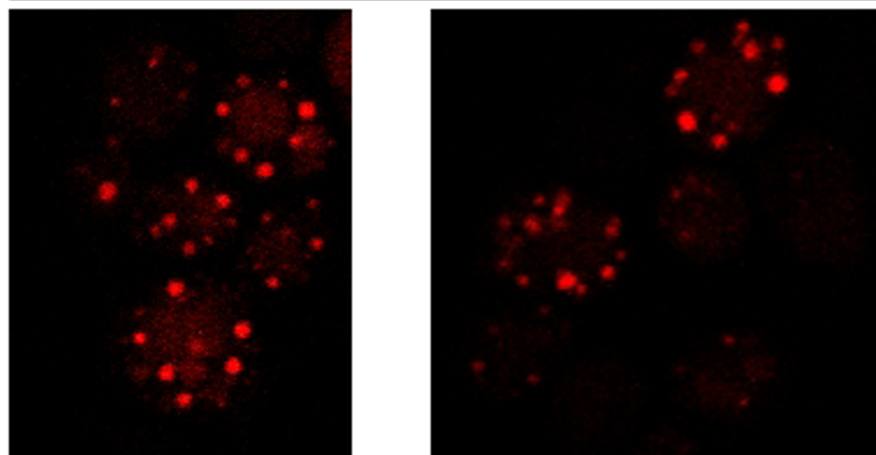

*[PIN<sup>+</sup>][psi<sup>-</sup> HSP104*

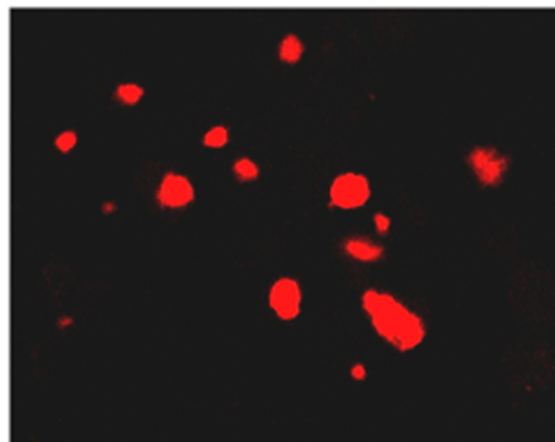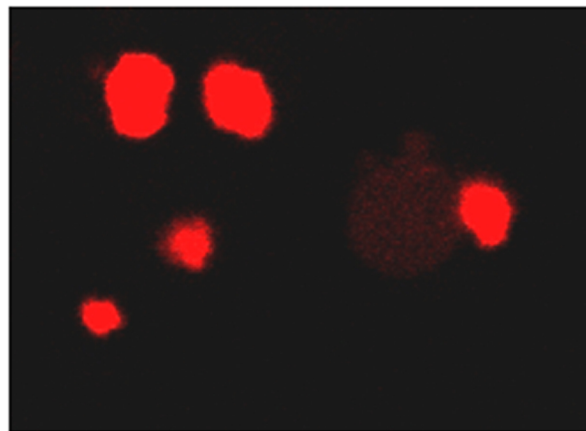

↑Sup35NM-GFP↑ Pin4C-RFP

Supplement: S12 Fig — Pin4C aggregates were not affected by the presence vs. absence of HSP104, but were larger in the presence of [PIN+]. Sup35NM-GFP and Pin4C-RFP were respectively co-overexpressed from p1181 and p1708, by growing hsp104Δ (L1802, or L1803), HSP104 [pin-] (L2910) or HSP104 [PIN+] (L1749) cells in 50 µM CuSO4 and 2% Gal. Pin4C-RFP aggregates were larger and more numerous in [PIN+] than [pin-] cells [134] but were the same in the presence or absence of Hsp104 in [pin-] cells. (PDF) [file pgen.1004814.s012.pdf]
