# Supplementary material for: Heterologous Aggregates Promote De Novo Prion Appearance via More than One Mechanism
Source: PLoS Genet. 2015 Jan 8;11(1):e1004814. doi: 10.1371/journal.pgen.1004814 (PMC4287349; doi:10.1371/journal.pgen.1004814)
Supplement: S4 Fig — Control experiments for colocalization of Sup35NM-YFP aggregates with Rnq1-CFP. A. Control [pin-] cells with integrated RNQ1-CFP and overexpressed Sup35NM-YFP. Sup35NM-YFP was overexpressed (p1753) in [pin-] RNQ1-CFP integrants. Both Rnq1-CFP and Sup35NM-YFP remained diffuse after 48 h. B. Control [PIN+] cells with integrated Rnq1-CFP and repressed Sup35NM-YFP. [PIN+] RNQ1-CFP integrants with the p1753 plasmid were grown in repressing media (Glucose) to inhibit Sup35NM-YFP overexpression. As expected, Rnq1-CFP formed dots, while Sup35NM-YFP displayed no signals after 48 h. C. Control [PIN+] cells with integrated Rnq1-CFP and overexpressed YFP. [PIN+] RNQ1-CFP integrants with the empty vector p1752 (YFP) were grown in 2% Gal. Rnq1-CFP showed only dots, indicative of [PIN+], while YFP always remained diffuse. (PDF) [file pgen.1004814.s004.pdf]

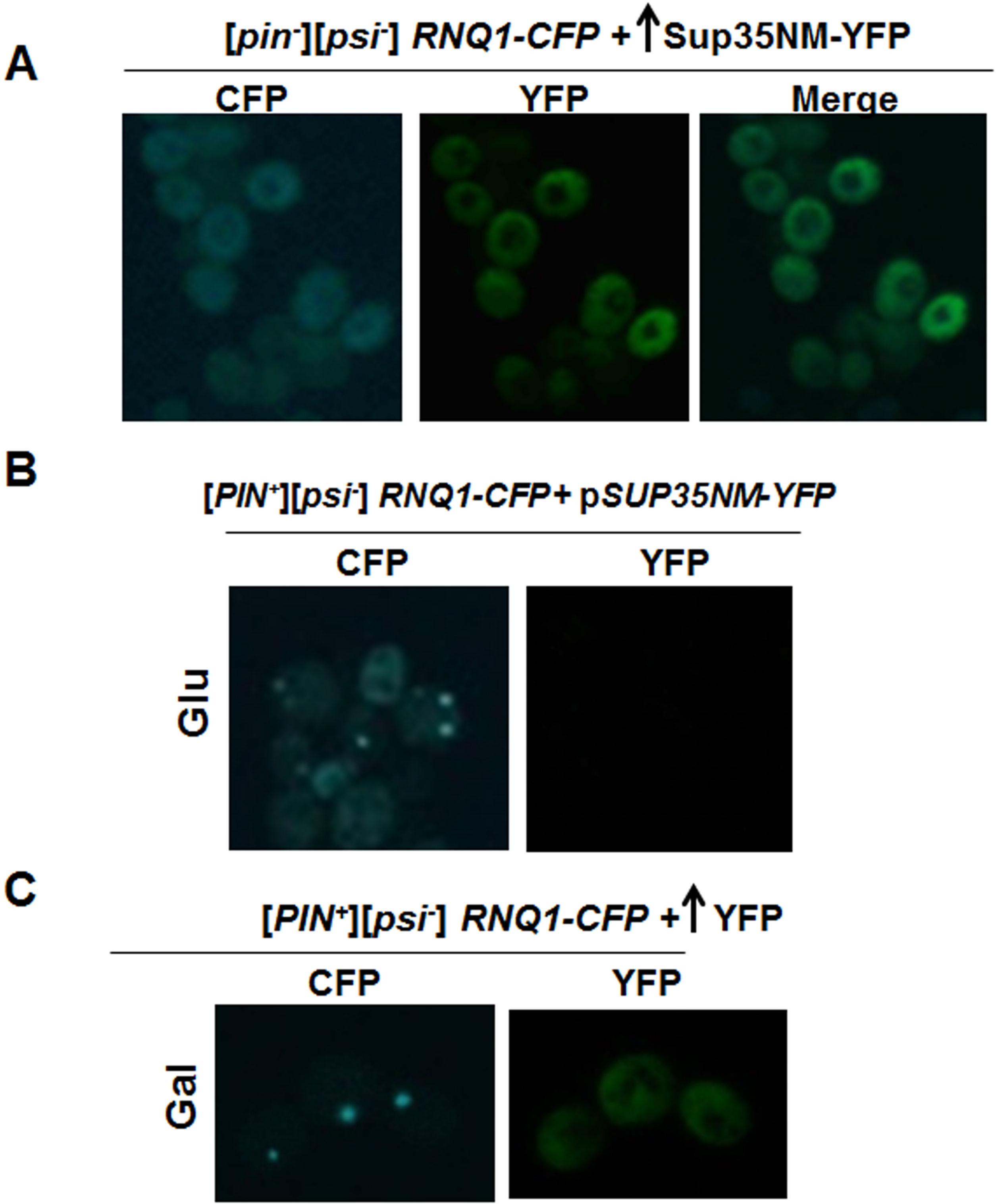
A
[pin^-][psi^-] RNQ1-CFP + ↑Sup35NM-YFP
CFP
YFP
Merge
B
[PIN^+][psi^-] RNQ1-CFP+ pSUP35NM-YFP
CFP
YFP
Glu
C
[PIN^+][psi^-] RNQ1-CFP +↑ YFP
CFP
YFP
Gal
