# Supplementary material for: Heterologous Aggregates Promote De Novo Prion Appearance via More than One Mechanism
Source: PLoS Genet. 2015 Jan 8;11(1):e1004814. doi: 10.1371/journal.pgen.1004814 (PMC4287349; doi:10.1371/journal.pgen.1004814)
Supplement: S6 Fig — Rnq1 and Sup35 form a close physical interaction during [PSI+< induction, but do not in established [PSI+]. Sup35NM-VN (p1893) and Rnq1-VC (p1894) in [PIN+][psi-] or [PIN+][PSI+] 74D-694 cells were co-overexpressed by growth in 0.2% Gal for the indicated times. (n≈600). Expression levels of Sup35NM-VN and Rnq1-VC (bottom) were detected by respectively, α-Sup35N and α-Rnq1 (a kind gift of S. Lindquist) in [PIN+][psi-] (left) or [PIN+][PSI+] (right) cells harboring p1893 and p1894, and grown in 0.2% Gal for 48 h. Pgk1 was used as an internal loading control. (PDF) [file pgen.1004814.s006.pdf]

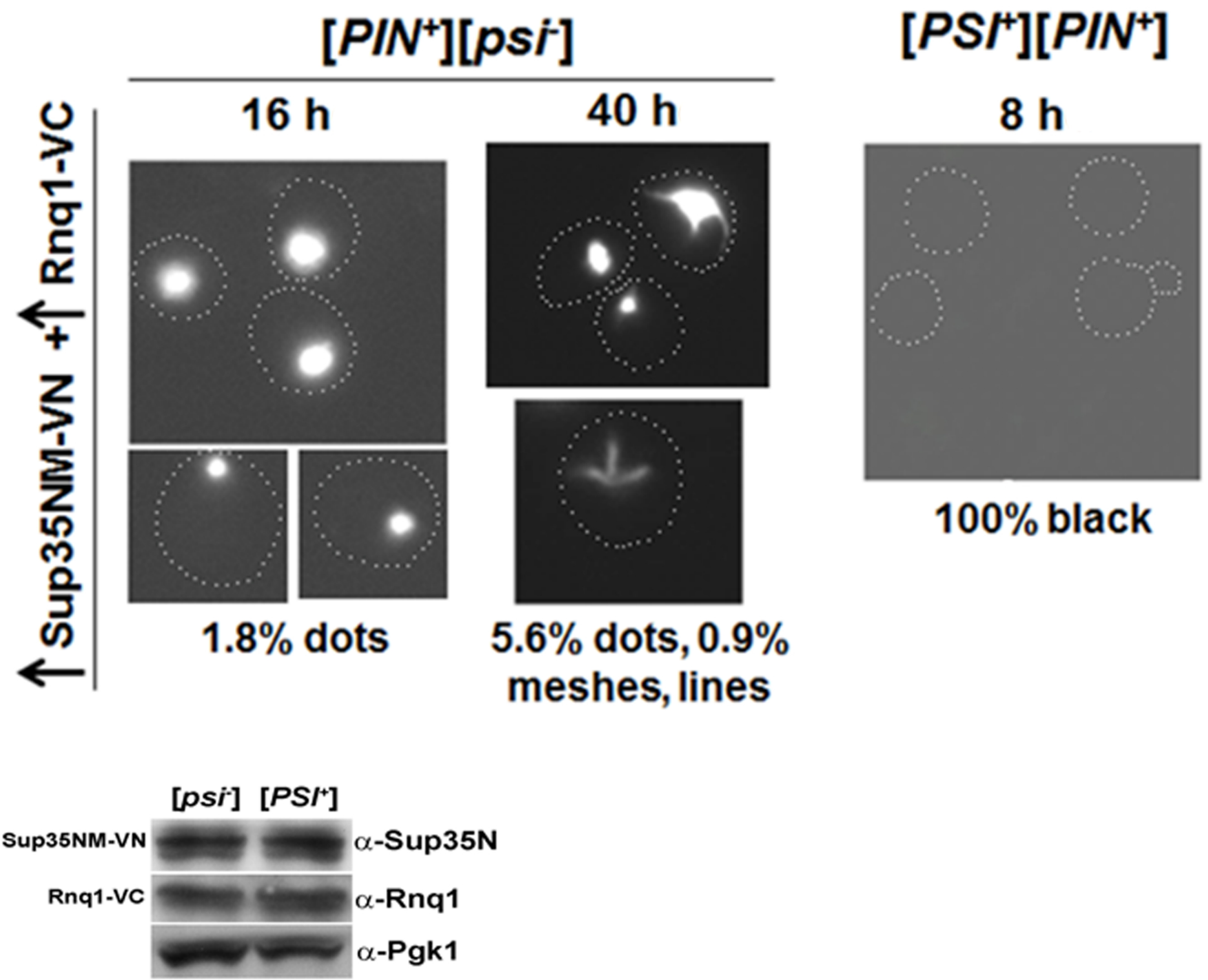

[PIN+][psi-]
[PSI+][PIN+]
16 h
40 h
8 h
Sup35NM-VN + Rnq1-VC
1.8% dots
5.6% dots, 0.9% meshes, lines
100% black
[psi-]
[PSI+]
Sup35NM-VN
Rnq1-VC
α-Sup35N
α-Rnq1
α-Pgk1
