# Supplementary material for: Heterologous Aggregates Promote De Novo Prion Appearance via More than One Mechanism
Source: PLoS Genet. 2015 Jan 8;11(1):e1004814. doi: 10.1371/journal.pgen.1004814 (PMC4287349; doi:10.1371/journal.pgen.1004814)
Supplement: S8 Fig — Colocalization of newly induced Sup35 aggregates with chaperones. A. Sup35 aggregates colocalized with Ssa1 and Sis1 chaperones. [PIN+] cells with GFP-tagged SSA1 or SIS1 endogenously were induced to overexpress Sup35NM-RFP (p2017) for 48 h by growth in 2% Gal. Observed Sup35NM-RFP rings in SSA1-GFP cells (5.6%, n≈450) completely colocalized with Ssa1-GFP. Sup35NM-RFP rings (5.5%, n≈400) completely colocalized with Sis1-GFP in SIS1-GFP cells. B. Hsp104 did not form aggregates in [pin-] cells. Overexpressed Sup35NM-RFP (p2017) in [pin-] HSP104-GFP cells with 2% Gal for 48 h resulted in no aggregate formation. (PDF) [file pgen.1004814.s008.pdf]

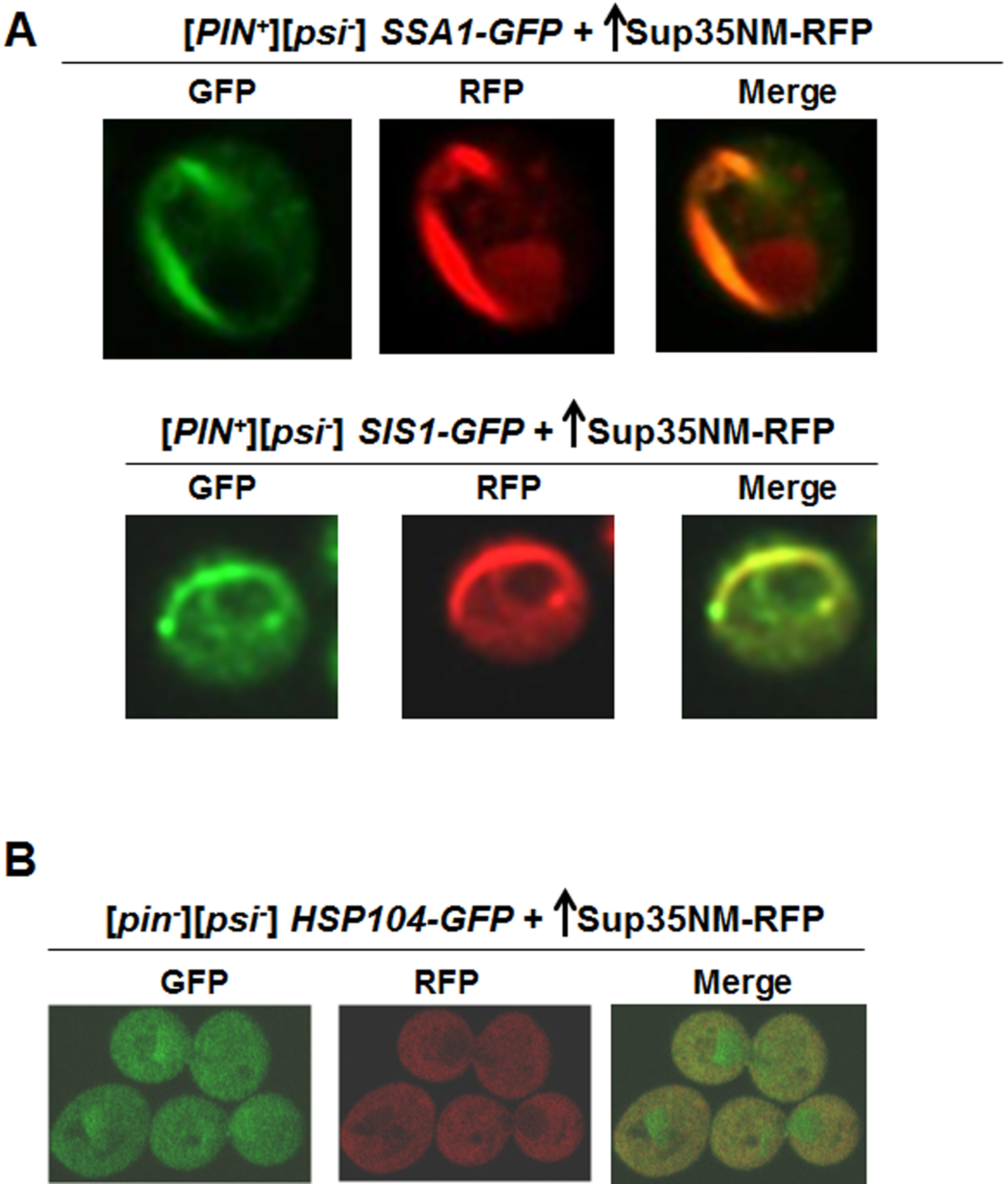
A
[PIN+][psi-] SSA1-GFP + ↑Sup35NM-RFP
GFP
RFP
Merge
[PIN+][psi-] SIS1-GFP + ↑Sup35NM-RFP
GFP
RFP
Merge
B
[pin-][psi-] HSP104-GFP + ↑Sup35NM-RFP
GFP
RFP
Merge
