# Supplementary material for: Heterologous Aggregates Promote De Novo Prion Appearance via More than One Mechanism
Source: PLoS Genet. 2015 Jan 8;11(1):e1004814. doi: 10.1371/journal.pgen.1004814 (PMC4287349; doi:10.1371/journal.pgen.1004814)
Supplement: S10 Fig — Colocalization of Pin4C-RFP with Sup35NM-GFP and Hsp42-GFP. A. Pin4C-RFP does not form rings in the absence of Sup35NM-GFP overexpression, and Sup35NM-GFP does not form aggregates without Pin4C overexpression. 74D-694 rnq1Δ cells, which contained either p1708 or 1951, were grown in 2% Gal to separately overexpress respectively, Pin4C-RFP or Sup35NM-GFP for 72 h. Pin4C-RFP formed large fluorescence dots, while Sup35NM-RFP remained diffuse. B. Sup35NM overexpression changes the location of Pin4C relative to the Hsp42-GFP dot. Pin4C-RFP was overexpressed from p1708 in [pin-] HSP42-GFP cells in the presence (top) or absence (bottom) of Sup35NM overexpression (from p1893-2). (PDF) [file pgen.1004814.s010.pdf]

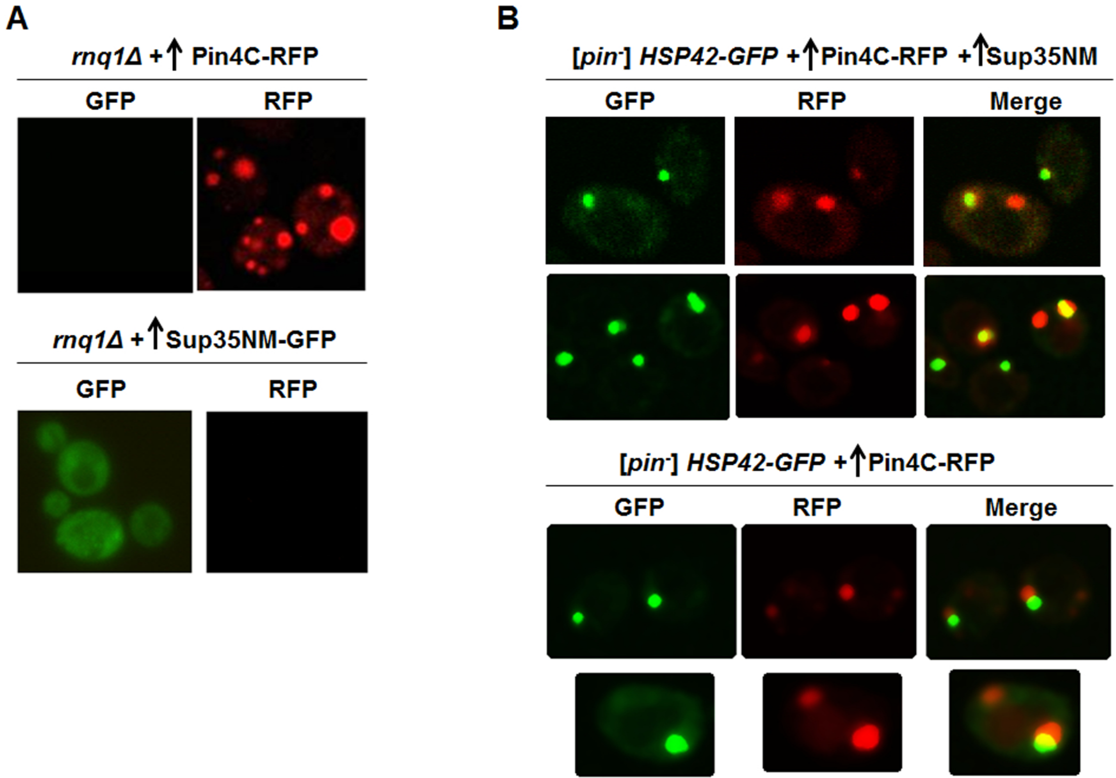

A
rnq1Δ +↑ Pin4C-RFP
GFP
RFP
rnq1Δ +↑Sup35NM-GFP
GFP
RFP
B
[pin-] HSP42-GFP +↑Pin4C-RFP +↑Sup35NM
GFP
RFP
Merge
[pin-] HSP42-GFP +↑Pin4C-RFP
GFP
RFP
Merge
