# Supplementary material for: Heterologous Aggregates Promote De Novo Prion Appearance via More than One Mechanism
Source: PLoS Genet. 2015 Jan 8;11(1):e1004814. doi: 10.1371/journal.pgen.1004814 (PMC4287349; doi:10.1371/journal.pgen.1004814)
Supplement: S1 Table — Visualization of Sup35NM-GFP aggregation in [PIN+] cells by growth in 0.2% Gal. Sup35NM-GFP in 74D-694 [PIN+][psi-] cells was overexpressed from p1951 by growth in 0.2% Gal. (PDF) [file pgen.1004814.s013.pdf]

Table S1. Visualization of Sup35NM-GFP aggregation in [*PIN*$^{+}$] cells by growth in 0.2% Gal.

**Time after addition of 0.2% Gal (h)**	**Sup35NM-GFP Fluorescence**
34	0.1% dots
48	0.1% peripheral rings/lines, 0.01% dots
70	0.1% dots, 0.5% peripheral rings/lines
