# Supplementary material for: Heterologous Aggregates Promote De Novo Prion Appearance via More than One Mechanism
Source: PLoS Genet. 2015 Jan 8;11(1):e1004814. doi: 10.1371/journal.pgen.1004814 (PMC4287349; doi:10.1371/journal.pgen.1004814)
Supplement: S2 Table — Colocalization data of Rnq1-GFP with Sup35-RFP after 6 h of induction of Sup35-RFP in [PIN+] cells. After 6 h of induction of Sup35-RFP (p1678) by growth of 74D-694 [PIN+][psi-] cells with p1730 expressing Rnq1-GFP from its own promoter in 2% Gal, 242 cells were seen to have Sup35-RFP dots out of 6000 cells counted. Among these 242 cells, 144 also showed Rnq1-GFP dots colocalized with Sup35-RFP, but the other 98 cells had diffuse Rnq1-GFP. (PDF) [file pgen.1004814.s014.pdf]

Table S2. Colocalization data of Rnq1-GFP with Sup35-RFP after 6 h of induction of Sup35-RFP in [PIN^+] cells.

**Total number of cells with Sup35-RFP dots (n=6000)**	**242 out of 6000 (4%)[a]**
Total number of cells with colocalizing Rnq1-GFP dots in cells with Sup35-RFP dots	144 out of 242 (60%)
Total number of cells with diffuse Rnq1-GFP in cells with Sup35-RFP dots	98 out of 242 (40%)

[a]Representative images are provided in Figure 4A.
