# Supplementary material for: Heterologous Aggregates Promote De Novo Prion Appearance via More than One Mechanism
Source: PLoS Genet. 2015 Jan 8;11(1):e1004814. doi: 10.1371/journal.pgen.1004814 (PMC4287349; doi:10.1371/journal.pgen.1004814)
Supplement: S3 Table — Colocalization data of Rnq1-GFP with Sup35-RFP after 24 h of induction of Sup35-RFP in [PIN+] cells. After 24 h of induction of Sup35-RFP (p1678) by growth of 74D-694 [PIN+][psi-] cells with p1730 expressing Rnq1-GFP on its own promoter in 2% Gal, 575 cells were seen to have Sup35-RFP lines/rings out of 4800 cells counted. Among these 575 cells, 401 also showed Rnq1-GFP rings/lines colocalized with Sup35-RFP rings/lines, while 57 had Rnq1-GFP dots and the other 117 had diffuse Rnq1-GFP. (PDF) [file pgen.1004814.s015.pdf]

Table S3. Colocalization data of Rnq1-GFP with Sup35-RFP after 24 h of induction of Sup35-RFP in [*PIN*$^+$] cells.

**Total number of cells with Sup35-RFP lines/rings (n=4800)**	**575 out of 4800 (12%)**
Total number of cells with Rnq1-GFP rings/lines in cells with Sup35-RFP rings/lines	401 out 575 (70%)
Total number of cells with Rnq1-GFP dots in cells with Sup35-RFP rings/lines	57 out of 575 (10%)[a]
Total number of cells with diffuse Rnq1-GFP in cells with Sup35-RFP rings/lines	117 out of 575 (20%)[a]

[a]Representative images are provided in Figure S3A.
