# Supplementary material for: Heterologous Aggregates Promote De Novo Prion Appearance via More than One Mechanism
Source: PLoS Genet. 2015 Jan 8;11(1):e1004814. doi: 10.1371/journal.pgen.1004814 (PMC4287349; doi:10.1371/journal.pgen.1004814)
Supplement: S4 Table — Colocalization data of Sup35-RFP with Rnq1-GFP after 24 h of induction of Sup35-RFP in [PIN+] cells. After 24 h of induction of Sup35-RFP (p1678) by growth of 74D-694 [PIN+][psi-] cells with p1730 expressing Rnq1-GFP on its own promoter in 2% Gal, 480 cells were seen to have Rnq1-GFP lines or rings out of 6000 cells counted. Among these 480 cells, 474 also showed Sup35-RFP rings/lines colocalized with Rnq1-GFP, but the other 6 cells had diffuse Sup35-RFP. (PDF) [file pgen.1004814.s016.pdf]

Table S4. Colocalization data of Sup35-RFP with Rnq1-GFP after 24 h of induction of Sup35-RFP in [*PIN*$^{+}$] cells.

**Total number of cells with Rnq1-GFP lines/rings (n=6000)**	**480 out of 6000 (8%)**
Total number of cells with Sup35-RFP rings/lines in cells with Rnq1-GFP rings/lines	474 out of 480 (99%)[a]
Total number of cells with diffuse Sup35-RFP in cells with Rnq1-GFP rings/lines	6 out of 480 (1%)

[a]Representative images are provided in Figure 4B.
