# Supplementary material for: Heterologous Aggregates Promote De Novo Prion Appearance via More than One Mechanism
Source: PLoS Genet. 2015 Jan 8;11(1):e1004814. doi: 10.1371/journal.pgen.1004814 (PMC4287349; doi:10.1371/journal.pgen.1004814)
Supplement: S5 Table — Colocalization data of Rnq1-CFP with Sup35NM-YFP after 24 h of induction of Sup35NM-YFP in [PIN+] cells. After 24 h of induction of Sup35NM-YFP (p1753) by growth of 74D-694 [PIN+][psi-] RNQ1-CFP cells with p1753 in 2% Gal, 63 cells were seen to have Sup35NM-YFP dots, and 7 cells had Sup35NM-YFP rings/lines out of 900 cells counted. Among these 63 dot-bearing cells, 57 also showed Rnq1-CFP dots colocalized with Sup35NM-YFP, while the other 6 cells had diffuse Rnq1-CFP. However, among the 7 Sup35-RFP ring bearing cells, they all had Rnq1-CFP rings colocalized with Sup35-RFP. (PDF) [file pgen.1004814.s017.pdf]

Table S5. Colocalization data of Rnq1-CFP with Sup35NM-YFP after 24 h of induction of Sup35NM-YFP in [*PIN*$^{+}$] cells.

**Total number of cells with Sup35NM-YFP dots (n=900)**	**63 out of 900 (7%)[a]**
Total number of cells with colocalized Rnq1-CFP dots in cells with Sup35NM-YFP dots	57 out 63 (90%)
Total number of cells with diffuse Rnq1-CFP in cells with Sup35NM-YFP dots	6 out of 63 (10%)
**Total number of cells with Sup35NM-YFP rings/lines (n=900)**	**7 out of 900 (0.8%)**
Total number of cells with colocalized Rnq1-CFP rings/lines in cells with Sup35NM-YFP rings/lines	7 out 7 (100%)

[a]Representative images are provided in Figure 4C, top.
