# Supplementary material for: Heterologous Aggregates Promote De Novo Prion Appearance via More than One Mechanism
Source: PLoS Genet. 2015 Jan 8;11(1):e1004814. doi: 10.1371/journal.pgen.1004814 (PMC4287349; doi:10.1371/journal.pgen.1004814)
Supplement: S6 Table — Colocalization data of Rnq1-CFP with Sup35NM-YFP after 48 h of induction of Sup35NM-YFP in [PIN+] cells. After 48 h of induction of Sup35NM-YFP (p1753) by growth of 74D-694 [PIN+][psi-] RNQ1-CFP cells with p1753 in 2% Gal, 216 cells were seen to have Sup35NM-YFP lines/rings out of 2000 cells counted. Among these 216 cells, 162 also showed Rnq1-CFP lines/rings colocalized with Sup35NM-YFP, while 15 harbored Rnq1-CFP dots and the other 39 had diffuse Rnq1-CFP. (PDF) [file pgen.1004814.s018.pdf]

Table S6. Colocalization data of Rnq1-CFP with Sup35NM-YFP after 48 h of induction of Sup35NM-YFP in [PIN^+] cells.

**Total number of cells with Sup35NM-YFP lines/rings (n=2000)**	**216 out of 2000 (11%)**
Total number of cells with Rnq1-CFP rings/lines in cells with Sup35NM-YFP rings/lines	162 out 216 (75%)[a]
Total number of cells with Rnq1-CFP dots in cells with Sup35NM-YFP rings/lines	15 out of 216 (7%)[a]
Total number of cells with diffuse Rnq1-CFP in cells with Sup35NM-YFP rings/lines	39 out of 216 (18%)[a]

[a]Representative images are provided in Figure S5.
