# Supplementary material for: Heterologous Aggregates Promote De Novo Prion Appearance via More than One Mechanism
Source: PLoS Genet. 2015 Jan 8;11(1):e1004814. doi: 10.1371/journal.pgen.1004814 (PMC4287349; doi:10.1371/journal.pgen.1004814)
Supplement: S7 Table — Colocalization data of Sup35NM-YFP with Rnq1-CFP after 48 h of induction of Sup35NM-YFP in [PIN+] cells. After 48 h of induction of Sup35NM-YFP (p1753) by growth of 74D-694 [PIN+][psi-] RNQ1-CFP cells with p1753 in 2% Gal, 375 cells were seen to have Rnq1-CFP lines or rings out of 5000 cells counted. Among these 375 cells, 371 also showed Sup35NM-YFP rings/lines colocalized with Rnq1-CFP, but the other 4 cells had diffuse Sup35NM-YFP. (PDF) [file pgen.1004814.s019.pdf]

Table S7. Colocalization data of Sup35NM-YFP with Rnq1-CFP after 48 h of induction of Sup35NM-YFP in [*PIN*$^{+}$] cells.

**Total number of cells with Rnq1-CFP lines/rings (n=5000)**	**375 out of 5000 (7.5%)**
Total number of cells with Sup35NM-YFP rings/lines in cells with Rnq1-CFP rings/lines	371 out of 375 (99%)
Total number of cells with diffuse Sup35NM-YFP in cells with Rnq1-CFP rings/lines	4 out of 375 (1%)
