# Supplementary material for: Heterologous Aggregates Promote De Novo Prion Appearance via More than One Mechanism
Source: PLoS Genet. 2015 Jan 8;11(1):e1004814. doi: 10.1371/journal.pgen.1004814 (PMC4287349; doi:10.1371/journal.pgen.1004814)
Supplement: S8 Table — Data for overexpressed Sup35 and Rnq1 from the BiFC constructs Sup35NM-VN and Rnq1-VC, respectively by growing cells in 0.2% Gala. Sup35NM-VN (p1893) and Rnq1-VC (p1894) in 74D-694 [PIN+][psi-] or [pin-][psi-] cells were co-overexpressed by growth in 0.2% Gal. The control [pin-] cells showed no fluorescence at any time. BiFC fluorescence was checked by YFP filter. Percentages are based on n≈600. (PDF) [file pgen.1004814.s020.pdf]

Table S8. Data for overexpressed Sup35 and Rnq1 from the BiFC constructs Sup35NM-VN and Rnq1-VC, respectively by growing cells in 0.2% Gal[a].

Time after addition of 0.2% Gal (h)	BiFC Fluorescence in [*PIN*$^+$][*psi*$^-$] cells	BiFC Fluorescence in [*pin*$^-$][*psi*$^-$] cells
16	1.8% dots	No fluorescence
24	4.0% dots	No fluorescence
40	5.6% dots, 0.9% peripheral rings/lines/meshes	No fluorescence

[a]Representative images are provided in Figure S6.
