# Supplementary material for: Heterologous Aggregates Promote De Novo Prion Appearance via More than One Mechanism
Source: PLoS Genet. 2015 Jan 8;11(1):e1004814. doi: 10.1371/journal.pgen.1004814 (PMC4287349; doi:10.1371/journal.pgen.1004814)
Supplement: S9 Table — Data of scoring for [PSI+] in rnq1Δ cells in the presence of Sup35NM and Pin4C overexpression. To see if observed Sup35NM-GFP aggregates in the absence of Rnq1 when Pin4C-RFP was overexpressed (p1708) were associated with the appearance of [PSI+] (Fig. 6), we scored cells grown in 2% Gal for different times for [PSI+] by color assay and growth on SD-Ade. Interestingly, cells taken after 16 h or less of induction with 2% Gal did not grow on SD-Ade at all, and were red on YPD. Cells induced for 24, 48, 72 h were able to grow on SD-Ade with a frequency of 2%, 2.9%, 5%, respectively. In addition, cells from the very same cultures with 24, 48, and 72 h of induction accumulated white/pink color when spread on YPD with a frequency of 1.5%, 3.2%, and 5.9%, respectively. (PDF) [file pgen.1004814.s021.pdf]

Table S9. Data of scoring for [PSI^+] in *rnq1Δ* cells in the presence of Sup35NM and Pin4C overexpression.

**Time after addition of 2% Gal (h)**	**# of white colonies (out of 1000 cells)[a]**	**# of Ade^+ colonies (out of 2000 cells)[b]**	**Phenotype of cells stained with Sup35NM-GFP[c]**
0	0	0	N/A
4	0	0	N/A
16	0	0	N/A
24	15 (1.5%)	40 (2%)	65% m.d.
48	32 (3.2%)	58 (2.9%)	60% m.d.
72	59 (5.9%)	99 (5%)	75% m.d.

a Cells taken at the indicated times were spread on YPD (1000 cells/plate) and the resulting white colonies were counted. (n=5 plates)

b Cells taken at indicated times were spread on SD-Ade (2000 cells/plate) and growing colonies were counted. (n=5 plates)

c Cells grown on SD-Ade, were then grown on 0.05% Gal for a few h to allow Sup35NM-GFP to decorate Sup35 aggregates. *m.d. multiple dots.* (n=20)
